# Basolateral Amygdala Input to the Claustrum: Topology, Excitatory Synapse Properties and Cellular Targets

**DOI:** 10.3390/cells15211937

**Published:** 2026-10-24

**Authors:** Gao-Xiang Ham, George J. Augustine

**Affiliations:** 1 Neuroscience & Mental Health Program, Lee Kong Chian School of Medicine, Nanyang Technological University, Singapore 308232, Singapore; gaoxiang90@gmail.com; 2 F.M. Kirby Neurobiological Center, Boston Children’s Hospital, Boston, MA 02115, USA; 3 Department of Molecular and Cellular Biology, Harvard University, Cambridge, MA 02138, USA; 4 Temasek Life Sciences Laboratory, Singapore 117604, Singapore

**Keywords:** claustrum, basolateral amygdala, functional connectivity

## Abstract

**Highlights:**

**What are the main findings?**

- Basolateral amygdala (BLA) input to the claustrum (CLA) is mostly excitatory and generates small synaptic responses compared to BLA input to neurons in the nearby insula or to cortical inputs to the CLA.
- While BLA axons preferentially overlap with CLA shell neurons more than CLA core neurons, and with dorsal CLA neurons projecting to the motor cortex more than CLA core neurons projecting to the retrosplenial cortex, functional innervation is uniform across all these regions.

**What are the implications of the main findings?**

- The BLA is functionally coupled to the CLA and likely provides an excitatory bias instead of being a direct driver of CLA neuron firing.
- There is only a loose correlation between macroscopic anatomy and physiological function for BLA inputs to the CLA.

**Abstract:**

Although the claustrum (CLA) has been increasingly implicated in salience and attentional processing, its relationship with the basolateral amygdala (BLA)—a key structure for emotional valence—remains poorly defined. Here, we used viral tracing, optogenetics, and brain slice electrophysiology to characterize the spatial organization and function of BLA–CLA circuitry. We found a clear anatomical compartmentalization of axonal projections between the BLA and CLA within the ring-like outer shell of the CLA, with little axonal labeling in the CLA core. Given this anatomical segregation, it was surprising that optogenetic photostimulation of BLA axons produced comparable excitatory responses in neurons in both the CLA core and shell. Pharmacological experiments indicated that BLA axons make monosynaptic contact with both shell and core neurons, with the latter likely doing so via their distal dendrites that extend into the shell. When CLA neurons were stratified according to their projection targets, BLA axons overlapped more with CLA neurons projecting to the motor cortex than those projecting to the retrosplenial cortex. However, synaptic responses to BLA input were similar in CLA neurons projecting to both cortical targets. Our results provide the first functional characterization of a BLA input pathway that potentially carries valence-related signals to the CLA. Because the excitatory synaptic responses produced in CLA neurons by BLA input were small and subthreshold for action potential generation, our results also argue that the BLA input contributes to a broader convergence of excitatory inputs instead of being a primary driver of CLA activity.

## 1. Introduction

The claustrum (CLA) has long fascinated neuroscientists because of its extensive connectivity with almost the entire cerebral cortex and with other brain regions [1,2,3]. Early work suggested that the CLA could be involved in multisensory integration [4]. However, since the landmark proposal by Crick and Koch that the CLA may serve as the seat of consciousness [5,6], most efforts have focused on the role of the CLA in cognition [1,7,8,9,10,11,12,13,14]. For example, behavioral analyses have implicated the CLA in salience detection [7,15], attention [13,16,17], impulsivity [18,19], learning and memory [12,20,21,22,23], anxiety [24], and sleep [25,26,27,28]. CLA function is highly specific and context-dependent: inhibiting the CLA selectively impairs performance when distractors are present or when associations are being learned but not during simple recall or undistracted performance [12,13]. Together, these findings suggest that the CLA helps select and prioritize information relevant for cognitive behavior.

The circuit mechanisms underlying the contributions of the CLA to cognitive function are poorly understood [29]. One major question is how limbic salience signals [30] are integrated into CLA circuits. Subcortical inputs to the CLA are relatively sparse but arise from key limbic structures—such as the mediodorsal thalamus (MD) and basolateral amygdala (BLA)—that are central to emotional valence and motivational salience [1,15,31,32,33]. This has given rise to the influential hypothesis that the CLA acts as an attention coordinator, integrating salience-related signals from regions such as the BLA and MD to guide cortical processing [1,15]. However, studies of the functions of pathways between subcortical structures to the CLA are scarce and are completely lacking for the BLA-to-CLA circuit.

Here, we combine viral tracing, optogenetics, and brain slice electrophysiology to define how BLA inputs engage claustral microcircuits and projection-defined subnetworks. We found that BLA inputs to the CLA are largely excitatory, but the resulting synaptic responses are small in amplitude and variable. Further, although we observed that BLA–CLA connectivity is anatomically biased toward the ring-like shell surrounding the PV-rich CLA core, excitatory BLA inputs are received by neurons present in both the shell and the core. We further demonstrate substantial anatomical overlap between BLA axons and CLA neurons projecting to both the motor cortex (M1) and the retrosplenial cortex (RSC), with similar functional BLA connectivity for both types of projection neuron. These results provide the first functional characterization of the BLA–CLA pathway and reveal a dissociation between structural topology and synaptic drive: anatomical imaging of input fiber density does not straightforwardly predict functional connectivity, particularly at the level of microcircuitry. Our results support a model in which BLA inputs do not act as the primary driver of CLA activity and instead provide a sparse, projection-biased salience signal that interacts with other cortical and subcortical inputs to shape how the CLA routes information to distinct cortical targets.

## 2. Materials and Methods

### 2.1. Animals

A total of 37 C56/BL6J wild-type male mice were used for the experiments (9 for anatomical tracing, 28 for electrophysiology). We employed only male mice to avoid potential complications due to estrous cycle variations in females. Mice used for tracing experiments were 2–3 months old at the time of virus injection, while mice used for the electrophysiological experiments were 1-2 months old at time of virus injection and 2–3 months old at the time of brain slice experiments. All animal procedures were performed according to the guidelines of the NTU Institutional Animal Care and Use Committee (protocol A18095, approved on 14 March 2019).

### 2.2. General Surgical Procedures for Viral Expression of Target Genes

To inject viruses into the brain, mice were deeply anesthetized using isoflurane and placed in a stereotaxic frame equipped with a heating pad. Animal condition was monitored via respiration and body temperature throughout surgery. For pre-surgery analgesic relief, buprenorphine and lidocaine were injected subcutaneously above the back and at the incision site, respectively. A skin incision was made, followed by a craniotomy and dura removal above the target coordinates. Precise viral injections were made using glass pipettes with tip diameters of 30–50 μm and a pressure injector (UltraMicroPump3 and SMARTouch Controller; World Precision Instruments, Sarasota, FL, USA). Both the glass pipettes and Hamilton syringes (5 μL model with RN Compression Fitting; Reno, NV, USA) were first filled with mineral oil before front filling with virus solutions. Viruses were injected at 1–2 nL/s into each target region. The coordinates used were as follows: RSC (ML: 0.5, AP: −2.5, DV: 0.8, angle: 8°), M1 (ML: −1.8, AP: 1.7, DV: −0.8), BLA (ML: −3, AP: −1.55, DV: −4.6), CLA (ML: −2.7, AP: 1.3, DV: −2.9). These coordinates are defined based on the following conventions of medial–lateral (ML) and anterior–posterior (AP), with depth based on dura (DV) while the angle represents the deflection from midline, with pipette tip pointing toward the center where applicable. After completing the injections, pressure was allowed to equilibrate for 5 min before retraction of the glass pipette, followed by incision suturing and recovery on a warming pad. Mice were monitored and supplied with an analgesic (Meloxicam) and an antibiotic (Baytril) in their drinking water for 1 week post-surgery.

### 2.3. Expression of Fluorescent Proteins/Opsins in BLA Axon Fibers and CLA Projection Neurons

To determine bidirectional neuroanatomical connectivity between CLA and BLA, an AAV cocktail was injected into the BLA. The cocktail contained equal parts of AAVretro-hSyn1-EGFP-iCre (the Viral Vector Facility of the Neuroscience Center Zurich; 100 nL; 10 × 10^12^ vg/mL) and AAV5-hsyn-ChrimsonR-tDT (University of North Carolina [UNC] vector core; 150 nL; 10 × 10^12^ vg/mL), which were used to label inputs to and outputs from the BLA, respectively.

To investigate BLA innervation of projection-specific CLA neurons, AAVs were injected into 2–3 different locations per animal. BLA output fibers were anterogradely labeled via injection of AAV5-hsyn-ChromsonR-tDt (UNC vector core) into the BLA, while projection-specific CLA neurons were retrogradely labeled via injection of AAVs (AAVretro-hSyn-EGFP-iCre or AAVretro-hSyn-EBFP-iCre; VVF-ZNZ) into M1 and/or RSC separately.

For the expression of opsins (ChrimsonR/ChETA) in BLA axons and presynaptic terminals, 150–250 nL of virus was injected into the BLA, using either AAV5/2-Syn-ChrimsonR-tdTomato (UNC Vector Core) or AAV5/2-Syn-ChETA-EGFP (made in-house). pAAV.hSynap.ChETA(E123T/H134R)-eYFP.WPRE.hGH was a gift from Karl Deisseroth (Addgene plasmid #100049; http://n2t.net/addgene:100049 (accessed on 15 August 2026; RRID:Addgene_100049). Fluorescent proteins (EGFP or mCherry) were paired with opsins labeled with contrasting fluorophores (ChrimsonR-tDT or ChETA-GFP) to aid post hoc identification of CLA somata and BLA fiber locations. Surgical procedures were identical to those described in Section 2.2.

For both anatomical and physiological experiments, the injection sites in each mouse were inspected to confirm that viruses were injected into the correct target regions. Animals in which fluorescence was not localized to the target regions were excluded from analysis. In our anatomical experiments, 5 of the 9 injections were made into the anterior BLA, and these resulted in the dense labeling of BLA axons in the CLA shell and dysgranular insula (DI) [34]. The remaining 4 mice did not have sufficient BLA axonal labeling within the CLA to be useful. For our physiological experiments, 25 out of 28 mice yielded slices with CLA neurons that exhibited synaptic responses in response to BLA photostimulation. Two of the remaining mice had weak expression of opsins in BLA fibers within the CLA and yielded no measurable light-evoked synaptic responses (4–5 cells examined). The third mouse was excluded because of poor brain slice quality, which prevented reliable recordings.

### 2.4. Histology and Immunohistochemistry

To allow adequate time for expression of fluorophores in anatomical experiments, animals were perfused and sacrificed 2–4 weeks post-surgery. Transcardial perfusion was performed using saline, followed by 4% paraformaldehyde (PFA) in phosphate-buffered saline (PBS). Dissected brains were post-fixed in 4% PFA overnight, followed by 30% sucrose solution in PBS for 2 days, or until the brains sank. Brain sections (60 µm thickness) were first washed, blocked, and then stained with primary antibodies—rabbit-anti-PV (1:1000, Swant: PV-28, RRID:AB_2315235), chicken-anti-GFP (1:1000, Abcam:AB_13970, RRID:AB_300798), and rat-anti-mCherry monoclonal antibody (16D7; 1:1000, from Thermo Fisher Scientific, RRID:AB_2536611)—overnight at room temperature (RT, 24 °C) or for 2 days at 4 °C. These sections were then washed and treated with secondary antibodies (1:1000, Alexa Fluor 488, Alexa Fluor 568, and Alexa Fluor 647; RRID:AB_142924, RRID:AB_2534121, RRID:AB_2536101) at RT for 2–3 h. Stained sections were also counterstained with DAPI to visualize cell nuclei.

### 2.5. Image Acquisition and Preprocessing

Images were acquired with a Zeiss Axio Scan.Z1 slide scanner (Carl Zeiss, Jena, Germany). To reduce out-of-focus blur, stained sections were imaged at different focal depths (using z-stack) in Extended Depth of Focus mode, which combines in-focus images from each z-plane to reconstruct a 2D projection. Tiled images were subsequently flat-field corrected and stitched using the WholeBrain program created by Daniel Fürth [35]. All images were processed using custom R scripts in RStudio (Version 2025.09.2 Build 418, Posit Software, PBC) and ImageJ macros (ImageJ version 1.54p / Fiji version 2.16.1) to rotate slices, crop CLA/AI regions, and separately process individual fluorescent channels. Locations of neuronal somata were quantified using processes adapted from the QUINT workflow [36]. Retrogradely labeled somata were segmented using Pixel classification in ilastik [37], followed by watershedding, particle exclusion (size: 60–infinity, circularity: 0.3–1), and binarization using ImageJ base functions. Anterograde-labeled axons were first extracted using a Hessian filter (FeatureJ version 2.0.0; https://imagescience.org/meijering/software/featurej/ (accessed on 15 August 2026); “largest eigenvalue of Hessian tensor” and “smoothing scale” factor set to 159), which enhanced the features of curvilinear fibers, followed by inversion and background subtraction (sliding paraboloid, 50-pixel radius = 33 µm) to enhance image contrast. PV-labeled sections were smoothed using a Gaussian Blur filter (sigma = 50) to homogenize fluorescence intensities to ease the identification of CLA and AI layers. The processed fluorescence channels were subsequently merged and quantified together.

### 2.6. CLA and AI Circuit Quantification

To quantify soma and fiber fluorescence across the CLA and AI layers, line profile measurements were made using ImageJ and subsequently organized and plotted using custom R scripts. Using the PV channel as a reference, for all AP sections, a line profile (200 µm in width) was drawn in ImageJ that spanned from the anterior commissure to the pia and crossed the CLA core (Figure S1A). A region of interest (ROI) was generated for each channel, and the profile plot was used to quantify the density of fluorescence across the ROI. Results from the profile plot were subsequently exported and further analyzed in R.

To aid cross-animal and cross-section comparisons, raw fluorescence values were smoothed to generate an average profile for each fluorescence channel. These average profiles were subsequently aligned across animals, relative to the anterior commissure, by using the first peak of PV labeling to identify the CLA core. AI layers were also identified based on the enrichment of PV expression in layers 2/3 and 5, while the lack of PV expression was used to identify the remaining layers. Due to a lack of markers for the CLA shell, the first fluorescence minimum following the peak fluorescence of the PV core was identified as the transition point between the CLA and the AI (also referred to as the CLA shell). Measurements from each channel were subsequently normalized for fluorescence intensity and compiled across animals based on the position of the AP. To ease interpretation, an average fit line was generated for each channel using local polynomial regression (locally estimated scatterplot smoothing, LOESS) to emphasize fluorescence differences across the CLA and AI layers. Statistical comparisons were performed using custom R scripts.

### 2.7. Brain Slice Electrophysiology

Acute brain slices were prepared 2–4 weeks after virus injection to allow sufficient time for opsin expression. Mice were anesthetized with isofluorane and perfused with ice-cold artificial cerebrospinal spinal fluid (ACSF) prior to euthanasia via decapitation. ACSF comprised the following: 126 mM NaCl, 24 mM NaHCO_3_, 1 mM NaH_2_PO_4_, 2.5 mM KCl, 2 mM CaCl_2_, 2 mM MgCl_2_, 10 mM glucose, and 0.4 mM ascorbic acid; 290–305 mOsm; pH 7.4; and gassed with 95% O_2_/5% CO_2_. Perfusion was done to clear blood vessels near the CLA. Brains were isolated and transferred into the cutting solution within 45 s to preserve slice viability. The cutting solution comprised the following: 250 mM sucrose, 26 mM NaHCO_3_, 10 mM glucose, 4 mM MgCl_2_, 3 mM myo-inositol, 2.5 mM KCl, 2 mM sodium pyruvate, 1.25 mM NaH_2_PO_4_, 0.5 mM ascorbic acid, 0.1 mM CaCl_2_, and 1 mM kynurenic acid; 350–360 mOsm; pH of 7.4; and gassed with 95% O_2_/5% CO_2_. Coronal brain slices (250 µm) were cut using a Leica VT 1000S vibratome; slices designated for patching were incubated at 34 °C in ACSF for 30 min to recover from cutting, while slices containing the virus injection site were immediately fixed in PFA for subsequent histological examination. Slices designated for patching were subsequently transferred to ACSF at RT and allowed to equilibrate for 20 min prior to recording.

Whole-cell patch clamp recordings were performed using techniques described in detail in Graf et al. [38]. In brief, recording electrodes (3.5–8.5 MΩ tip resistance) were pulled from borosilicate glass and filled with K-gluconate-based internal solution containing the following: 130 mM K-gluconate, 10 mM KOH, 2.5 mM MgCl_2_, 10 mM HEPES, 4 mM Na_2_ATP, 0.4 mM Na_3_GTP, 5 mM EGTA, 5 mM Na_2_ phosphocreatinin, 0.2% neurobiotin, and/or Alexa 594 (285–295 mOsm, pH 7.4). Brain slices were submerged in a recording chamber that was continuously perfused with oxygenated ACSF at room temperature. The recording pipettes were positioned visually using a combination of the differential interference contrast and fluorescence channels of a two-photon microscope (Olympus FV-1000, Olympus Corporation, Tokyo, Japan; 950 nm excitation wavelength). Recordings were excluded from analysis if the electrode access resistance was greater than 30 MΩ, or if the holding current was larger than 100 pA. Electrophysiological data were acquired at 50 kHz and filtered at 10 kHz using a Multiclamp 700B amplifier (Molecular Devices, San Jose, CA, USA) and a Digidata 1440 interface (Molecular Devices).

Patched neurons were electrophysiologically identified by recording their responses to 1 s long current pulses that increased in amplitude at 20 pA increments. From these responses, 21 intrinsic electrical properties were extracted [38] by using code adapted from the Claustrum Classifier: https://github.com/adityanairneuro/claustrum (accessed on 15 August 2026). These properties were extracted from voltage responses to 3 different current intensities: action potential (AP) threshold current (C_t_), a current twice as large as C_t_ (C_t_*2), and the current level where the maximum number of APs was evoked (C_max_). A brief description of these properties follows. The resting membrane potential and membrane resistance are characteristic passive membrane properties. Most other properties were calculated based on either the AP responses evoked by C_t_*2 or by C_max_. The coefficient of variance (CV) is commonly used as a measure of firing variability; the larger CV, the more irregular AP firing is. CV was calculated as the ratio of the standard deviation of interspike intervals (ISIs) divided by the mean ISI. Since doublet spiking was observed in a large portion of neurons—a typical feature of CLA projection neurons [38]—two additional CV values were calculated, CV-1 and CV-2, which excluded the first action potential and the first two APs, respectively. The amplitude ratio of the first AP over the second AP, the second AP over the third AP, and the first AP/last AP were also taken to represent the AP amplitude adaptation that are often observed in CLA neuron responses to prolonged depolarization [38]. Extracted properties were compiled and analyzed using custom R scripts and used to identify neurons, either as projection neurons (PN1–5) or interneurons (PV, SST, and VIP) [38].

### 2.8. Recording Synaptic Responses to Optogenetic Photostimulation

BLA fibers expressing ChrimsonR or ChETA were photostimulated with light (denoted as hν in figures) from a mercury arc lamp delivered to the specimen via a GFP filter cube (470–495 nm excitation; 505 nm dichroic mirror) or a 436/514/604 nm dichroic mirror, which delivered light at 473–515 nm and 550–607 nm. The former provided 45 mW of light at 500 nm (to activate ChETA), while the latter provided a similar amount of light power at 590 nm (to activate ChrimsonR). The entire specimen field (0.24 mm^2^ area) was illuminated with light flashes, whose duration (4 ms) was controlled by an electronic shutter (Uniblitz).

Whole-cell patch clamp recordings were used to detect light-evoked postsynaptic currents under voltage-clamp conditions. These responses were averaged across 25 trials, with a 5 s interval between each trial to allow time for any short-term synaptic plasticity evoked by a prior light flash to subside. Reported voltages are not corrected for liquid–liquid junction potentials. Current responses were pre-processed using Clampfit software (Version 11.4.3, part of the pCLAMP 11 Software Suite, Molecular Devices; RRID:SCR_011323): traces were filtered using a low-pass (500 Hz) Gaussian filter and were baseline-adjusted by subtracting the mean current measured 50 ms before the light flash. All further analysis was done using custom scripts written in R. Light-induced synaptic currents were analyzed between 5 and 25 ms post-stimulation. To exclude spontaneous synaptic currents and noisy responses, individual responses were first automatically detected using two criteria: (1) peak time was later than the mean time of current onset and less than the sum of mean current decay time plus mean current peak time; and (2) response amplitude was greater than 5 pA. Each response was then visually inspected to confirm that it exhibited time-locked behavior and a normal waveform. Response percentage was calculated as the number of trials with synaptic responses that met these criteria, divided by the total number of trials. The 5–25 ms time window is sufficiently wide to detect delayed synaptic events associated with jitter in presynaptic photostimulation.

In a subset of experiments, light-evoked synaptic potential responses were also recorded under current-clamp conditions. Synaptic potentials were recorded in response to trains of light flashes (2 s duration, each flash 4 ms in duration) during widefield photo -stimulation at 5 Hz and 10 Hz. Synaptic potentials were averaged across at least 10 trials with 4 s intervals between trials.

A subset of cells exhibiting synaptic responses to BLA photostimulation were further tested for monosynaptic inputs [39,40]. In these experiments, tetrodotoxin (TTX; 1 µM), a voltage-gated sodium channel blocker, was used to prevent action potentials evoked by photostimulation. This was followed by treatment with saline containing both TTX and 4-aminopyridine (4AP; 100 µM) [39,40] for at least 10 min. A subset of TTX+4AP experiments were performed under increased extracellular Ca^2+^ conditions (4 mM instead of the usual 2 mM), to increase EPSC amplitude and improve the likelihood of detecting the small responses typically observed in TTX+4AP conditions.

### 2.9. Post-Electrophysiology Immunohistochemistry

To visualize the location of patched cells via their neurobiotin labeling, as well as the PV and fluorescent proteins used to determine the location of the CLA and its BLA inputs, brain slices were fixed in PFA overnight at 4 °C and then processed via standard immunohistochemical processing. In short, slices were rinsed with PBS, blocked, and then permeabilized in PBS with 0.25% Triton X-100 and 5% goat-serum for an hour, followed by incubation in primary antibodies for 48 h at 4 °C (rabbit-anti-PV, 1:800, Swant: PV-28, RRID:AB_2315235; chicken-anti-GFP, 1:1000, Abcam: ab13970, RRID:AB_300798; rat-anti-mCherry, 1:1000, Thermo Fisher Scientific Cat# M11217, RRID:AB_2536611; and fluorophore-conjugated streptavidin, 1:1000, Alexa Fluor 405, or Alexa Fluor 647, Thermo Fisher Scientific). Slices were washed again and incubated in secondary antibodies for 2–3 h at room temperature (1:1000, Alexa Fluor 488, Alexa Fluor 568, or Alexa Fluor 647; RRID:AB_142924, RRID: AB_2534121, RRID: AB_2866492, Thermo Fisher Scientific), followed by a final wash, and mounted onto slides with an anti-fading agent (PVA, 10981, Sigma Aldrich). Images were acquired on a confocal microscope (Zeiss LSM 800, Carl Zeiss, Jena, Germany) using a 10x objective.

The locations of patched cells were first identified and labeled on post hoc stained images via Zeiss software (Zen Blue 3.2). Correlation of electrophysiological responses with neuron locations was done using a combination of live slice images and post hoc staining images. A custom ImageJ macro script was developed to further process the images for unbiased identification of the claustrum core and quantification of the location of patched cells. In brief, images from all channels were background-subtracted (Rolling Ball, 25 pixels (= 16.5 μm) radius, sliding paraboloid) and filtered (Gaussian Blur, 4 µm sigma). Each fluorescence channel was then binarized using different auto-thresholding methods. For retrogradely labeled CLA cells, Otsu was used [41], while thresholding of BLA fibers and PV staining was done with Yen [42]. The claustrum core was identified by enrichment of PV staining, and an ellipse was fitted to the shape of the CLA core. Our thresholding method was reliable in most cases and only minor adjustments had to be made in a subset of slices where PV cell enrichment did not form a complete ellipse. In these cases, the BLA enrichment zone was referenced to ensure that the size of the PV ellipse was accurate. ROIs for the CLA core and patched cell locations were added, quantified, and exported. Further analysis using the location information extracted from ImageJ was done using custom R scripts. In summary, assignment of the CLA border was made purely based on PV and BLA fiber enrichment and was independent of physiological responses or locations of patched cells.

### 2.10. Statistical Analysis

Because multiple neurons were often examined in the same animal, comparisons of most electrophysiological properties were performed using linear mixed-effects models (lme4, lmerTest [43,44]) with animal as a random intercept; comparisons of binary outcomes (e.g., response probability) instead used generalized linear mixed-effects models with a binomial link. Pairwise contrasts, 95% confidence intervals, and Cohen’s d effect sizes were obtained using emmeans and effectsize functions [45], following established recommendations for nested neuroscientific data [46]. Where too few mice were available to estimate a random-effects variance, results are reported descriptively instead. *p*-values were corrected for multiple comparisons within each panel of properties using the Benjamini–Hochberg procedure [47].

## 3. Results

### 3.1. Anatomy of Claustrum–Basolateral Amygdala Connectivity

**Figure 1:**
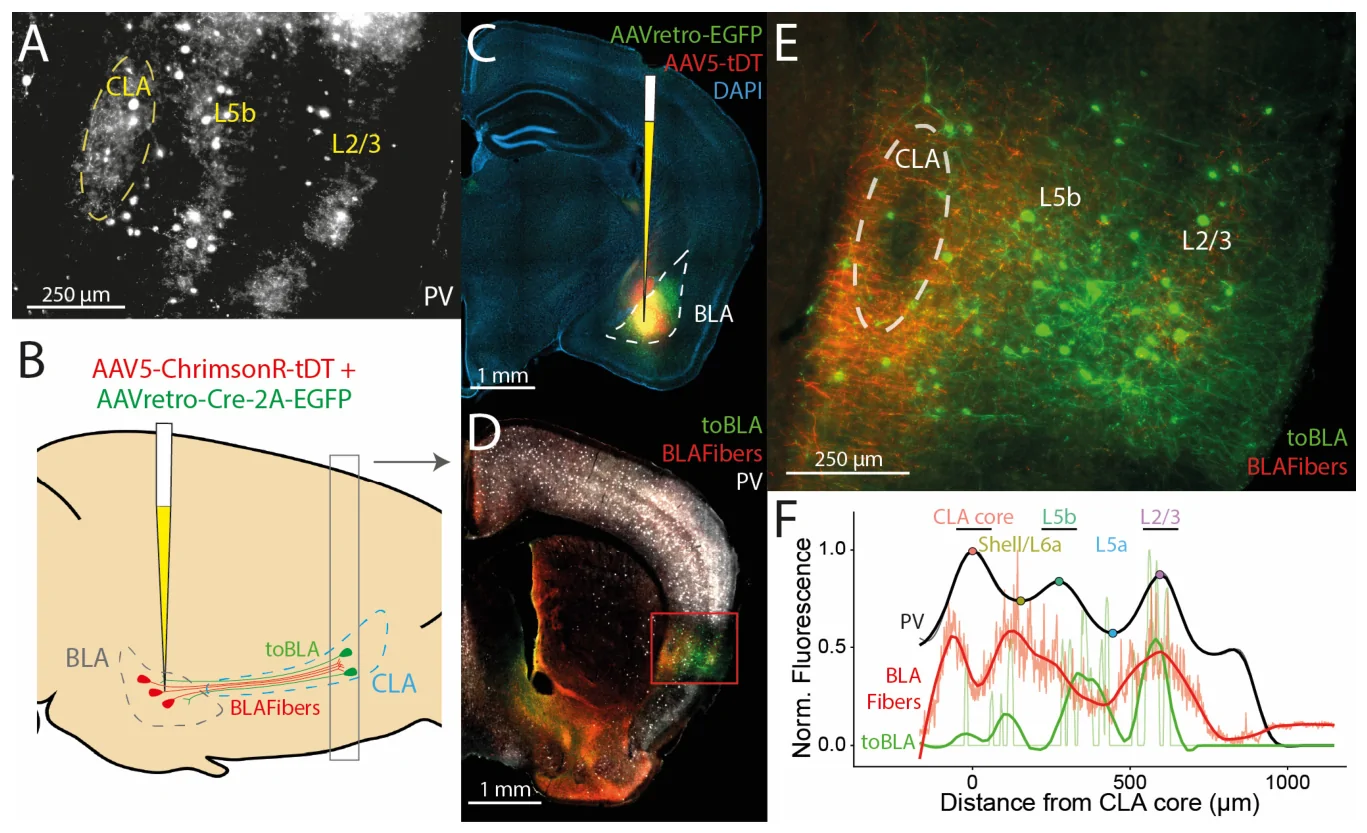
Anterograde and retrograde tracing reveals anatomical connectivity between BLA and CLA. (**A**) Parvalbumin (PV) is enriched in the CLA core and in agranular insula (AI) layers 5b (L5b) and 2/3 (L2/3). CLA core defined by PV immunoreactivity, as indicated by the dashed yellow line. (**B**) Schematic of viral tracing strategy and resulting fluorophore expression in BLA and CLA neurons. A cocktail of anterograde and retrograde AAV tracers was injected into the BLA to label BLA→CLA axons (*BLAFibers*, red) and CLA→BLA neurons (*toBLA*, green). The main image indicates a sagittal plane, while the gray rectangle depicts the coronal section shown in (**D**). (**C**) DAPI-stained coronal section showing tracer expression at the BLA injection site; dashed white line indicates the BLA. (**D**) PV-stained (white) coronal section showing BLA fibers (*BLAFibers*, red) and neurons projecting to BLA (*toBLA*, green). The red rectangle indicates the region magnified in (**E**). (**E**) Magnified two-channel image, with the white dashed line indicating the CLA core (from PV labeling). *toBLA* (green) neurons are enriched in AI but sparse in the CLA core. *BLAFibers* (red) avoid the CLA core and densely innervate the surrounding shell and AI L2/3. (**F**) Fluorescence intensity profiles across the CLA and AI. Values derived from this area are depicted by a yellow rectangle in Figure S1A. Bold lines show LOESS-smoothed averages; background lines show raw values. PV (black) was used to define CLA and AI layers. Colored points mark regions assigned by PV expression and are indicated by labels of the same color.

We first examined the anatomical organization of connections between the BLA and the CLA. The lack of an external capsule has led to inconsistent structural definitions of CLA boundaries in rodents. We employed the most widely used criterion for labeling the CLA core: post hoc parvalbumin (PV) immunohistochemistry [12,31,48,49]. This allowed us to visualize the PV-rich CLA core as an oval region (Figure 1A), as well as the strongly labeled PV interneurons in layers 5b and 2/3 of the nearby agranular insular cortex (AI; Figure 1A).

#### 3.1.1. Tracing Reveals BLA Axons and BLA-Projecting Neurons in the CLA Shell

To map the topography of BLA–CLA connectivity, we injected a mixture of retrograde and anterograde AAVs into the BLA (Figure 1B,C) and imaged the spatial distribution of the fluorophores expressed by these viruses in coronal sections that included the CLA (rectangle in Figure 1B). The retrograde virus (AAVretro-hsyn-Cre-2A-EGFP) produced a cytoplasmic GFP label in CLA neurons projecting to the BLA (*toBLA*; Figure 1D,E and Figure S1A—left), while the anterograde virus (AAV5-hsyn-ChrimsonR-TdT) labeled CLA-projecting BLA axons with membrane-bound tdTomato (*BLAFibers*; Figure 1D,E and Figure S1A—right).

We observed a relative absence of both EGFP (*toBLA*, green) and tdTomato (*BLAFibers*, red) within the CLA core (white dashed line; Figure 1E). Instead, both fluorophores were enriched in the region immediately surrounding the core, forming a ring-like structure (Figure 1D,E). This distinctive positioning is consistent with a location within the claustrum shell (CLA shell) [3,9,50,51,52]. For inter-animal comparison and objective delineation of the CLA, we masked retrogradely labeled CLA somata (*toBLA*, green) and anterogradely labeled BLA axonal fibers (*BLAFibers*, red) and sampled fluorescence from the anterior commissure to the pia across the CLA center (Figure S1A). Raw density profiles were smoothed to generate average spatial profiles (Figure 1F, bold lines). Profiles were centered on the first peak of PV staining (CLA core) from the anterior commissure; subsequent peaks were assigned to AI L5b and L2/3 (Figure 1F). Lacking a molecular marker for the CLA shell, we defined the first trough of PV staining as the boundary between the CLA and AI (shell/L6a).

#### 3.1.2. CLA Shell Neurons Project to the BLA

Retrograde tracing revealed an unexpected paucity of anatomical connectivity between the PV-rich CLA core and the BLA (Figure 1D, *toBLA*). Most *toBLA* neurons resided in upper AI layers, with only sparse labeling near the CLA core (Figure 1E). EGFP fluorescence was bright in upper-layer AI somata and neurites, whereas the neurites of CLA core neurons were weakly labeled. To focus on cell body locations, we segmented only *toBLA* somata. The fluorescence of masked *toBLA* somata was quantified from the anterior commissure to the pia, across the CLA core (Figure S1A—bottom, green), and smoothed to obtain average fluorescence spatial profiles (Figure 1F, *toBLA*). These analyses revealed a small number of *toBLA* neurons near the CLA shell/AI L6a but few neurons within the CLA core (Figure 1F). This paucity of *toBLA* neurons in the CLA core was consistent along the rostral–caudal axis in all mice, as reflected by low *toBLA* densities at the first PV peak (CLA core; Figure S1C, vertical line). A thin halo of *toBLA* neurons surrounded the CLA core along the entire rostral–caudal axis, appearing as narrow density peaks flanking the core that decreased with distance, in parallel with the shrinking CLA core (Figure S1C, background peaks). Our results indicate that BLA-projecting neurons largely avoid the PV-rich CLA core and preferentially occupy its border. This positioning of *toBLA* neurons suggests topological heterogeneity of neurons within the CLA complex according to their projection targets. In addition, many BLA-projecting neurons were located in L5a and L2/3 of the AI (Figure 1E, *toBLA*; Figure S1B,C), suggesting that AI-to-BLA connectivity could be greater than CLA-to-BLA connectivity.

#### 3.1.3. BLA Fibers Innervate a Ring-like CLA Shell and Avoid the CLA Core

CLA-based retrograde transsynaptic labeling indicates that BLA neurons project to the CLA [13,31], but it is unknown whether BLA axons innervate the entire CLA or only specific subregions. To resolve the structural organization of BLA inputs, we examined the distribution of anterogradely labeled BLA axons within the CLA (*BLAFibers*). *BLAFibers* largely avoided the CLA core and, instead, densely innervated the surrounding CLA shell (Figure 1E, *BLAFibers*). To quantify *BLAFibers* across animals, we masked axonal fluorescence and sampled fiber density across the CLA (red in Figure S1A—bottom), then generated smoothed average plots from raw measurements (Figure 1F, *BLAFibers*). *BLAFibers* density was highest in the CLA shell and decreased toward AI L5b (Figure 1F). The ring-like innervation pattern—two flanking density peaks that scaled with CLA size—was consistent along the rostral–caudal axis in all mice (Figure S1B,D). These observations indicate that BLA input preferentially innervates the CLA shell, though our functional analyses described next also surprisingly reveal a substantial BLA input to the CLA core.

### 3.2. Functional Connectivity Between the Basolateral Amygdala and Claustrum

Although the BLA-to-CLA pathway features prominently in hypotheses of CLA function [15], the functional properties of this pathway are unknown. Therefore, we next combined electrophysiology and optogenetics to determine the function of BLA-to-CLA projections. Further, because our tracing data indicated a topological bias for BLA axons within the CLA shell rather than the CLA core, we compared BLA synaptic input to neurons within the CLA core versus neurons within the CLA shell.

#### 3.2.1. Functional Properties of BLA Inputs to the CLA

**Figure 2:**
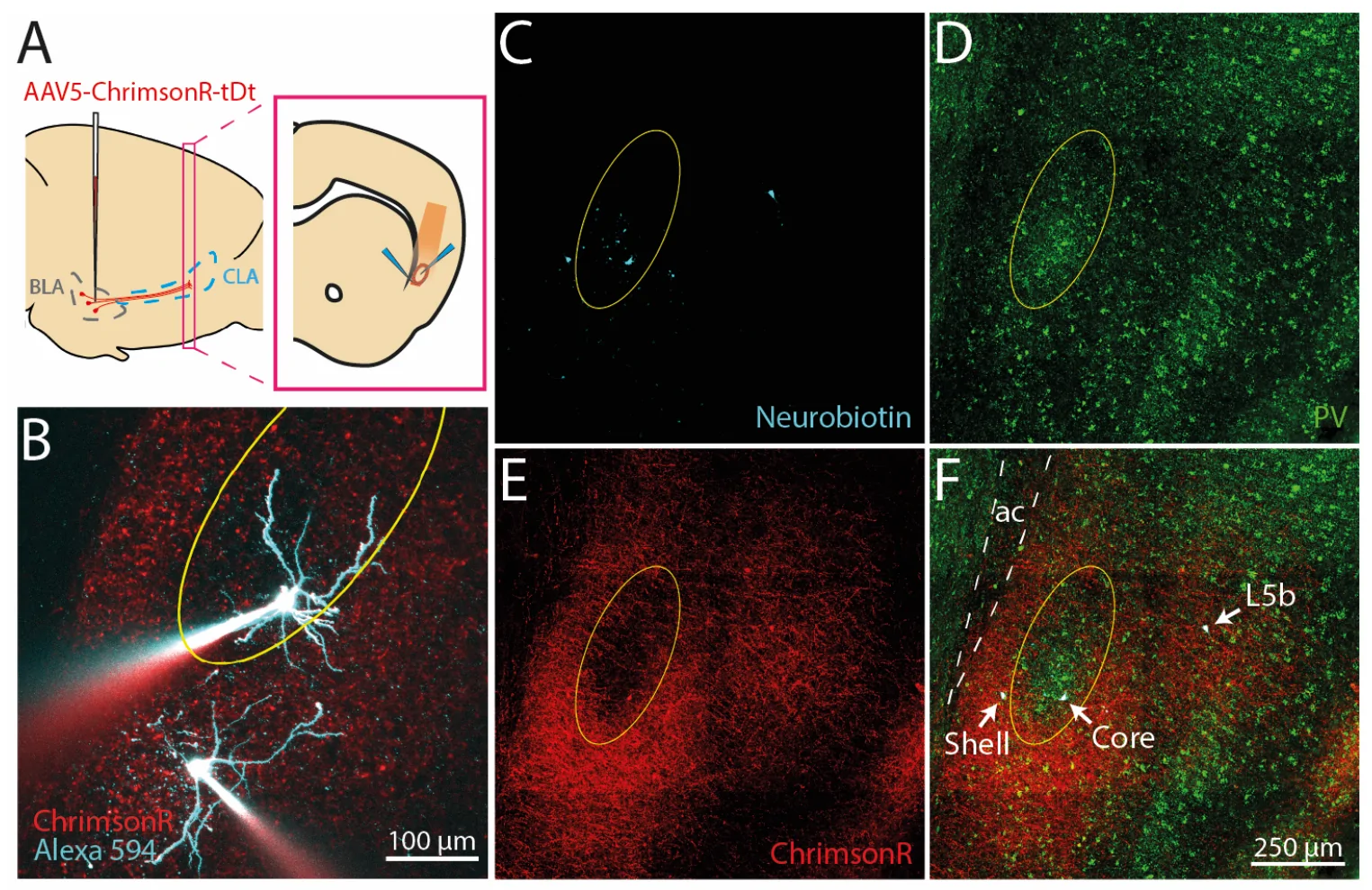
Experimental strategy for characterizing BLA synaptic inputs onto CLA neurons. (**A**) Left: Anterograde virus was injected into BLA to express ChrimsonR in BLA axons terminating in CLA. Right: Whole-cell patch clamp recordings in CLA were combined with optogenetic photostimulation of BLA fibers; blue triangles depict patch pipettes, and the orange gradient depicts photostimulation light. (**B**) Image of CLA in a living slice, with paired recordings from Alexa-filled neurons (cyan) in the CLA core and CLA shell. The pattern of ChrimsonR-expressing BLA fibers (red) was used to locate the CLA core (yellow ellipse). (**C**–**F**) Post hoc identification of patched neuron location after fixation. Yellow ellipse again depicts the CLA core. (**C**) Post hoc staining of neurobiotin-filled neurons (cyan) identified the location of somata of three patched cells. (**D**) Parvalbumin staining (green) used to identify CLA core and AI layers. (**E**) ChrimsonR-expressing BLA fibers (red) formed a ring around the CLA core. (**F**) Superimposition of images from (**C**–**E**). Labeled arrows indicate locations of three patched cells; ac indicates location of the anterior commissure.

To define the function of BLA-to-CLA projections, we combined optogenetic activation of BLA inputs with whole-cell patch-clamp recordings of synaptic responses of CLA neurons. The red-shifted opsin, ChrimsonR, was expressed in BLA neurons using an anterograde AAV, allowing selective photostimulation of BLA inputs when light was delivered to the CLA (Figure 2A, right). Synaptic connectivity between the BLA and the CLA was then assessed in coronal brain slices by measuring postsynaptic responses evoked in CLA neurons in response to widefield (0.24 mm^2^ area) photostimulation of BLA axons within the CLA (Figure 2A, inset). Recordings were obtained from a total of 89 projection neurons and 11 interneurons, as determined from their intrinsic electrical properties [38]. Neurons in both the CLA core and shell were examined. In some cases, both CLA core and shell neurons were patched simultaneously to allow direct comparison of BLA input properties (Figure 2B). Patched neurons were also filled with neurobiotin, which diffused from the recording pipette into their cytoplasm. This allowed us to examine neuronal structure (Figure 2B) and to determine the precise location of neurons (Figure 2C) relative to the PV-defined CLA core (Figure 2D) and to the distribution of ChrimsonR-expressing BLA axons (Figure 2E,F).

#### 3.2.2. Only a Subset of CLA Neurons Receive Excitatory Input from the BLA

**Figure 3:**
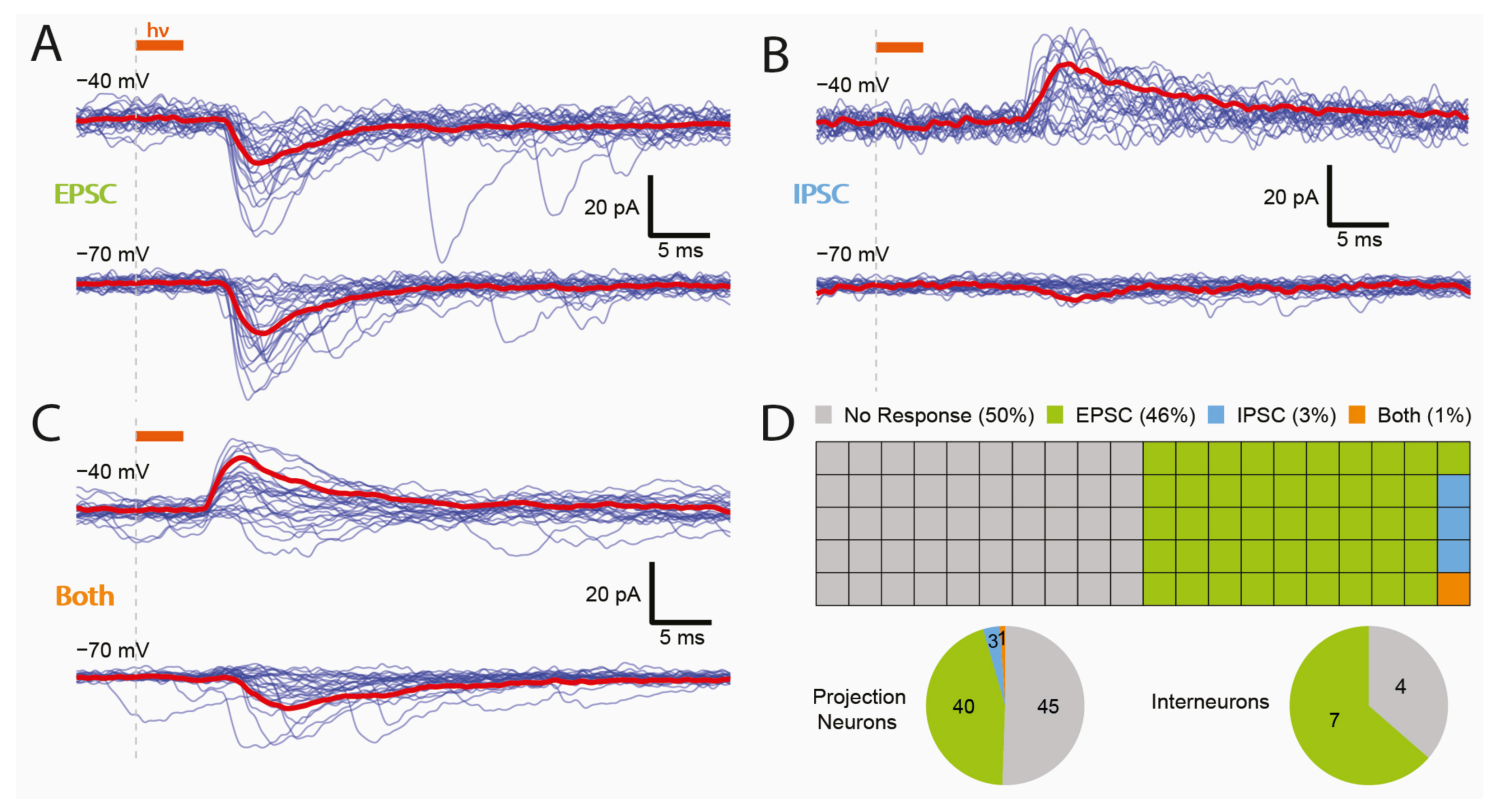
BLA synaptic inputs to CLA neurons. Neurons were held at potentials of −40 mV (top traces) and −70 mV (bottom traces). Orange bars represent the timing of light flashes (hν, 4 ms duration) used to photostimulate BLA axons. Blue lines represent individual traces, while red lines depict average postsynaptic currents. (**A**) Light-induced currents responses from a CLA neuron receiving excitatory synaptic input from the BLA; the presence of inward currents at both holding potentials indicates that the responses were EPSCs. (**B**) Light-induced current responses from a CLA neuron exhibiting inhibitory synaptic input from the BLA; IPSC responses could be identified because they were only observed at −40 mV. (**C**) Light-induced current responses from CLA neuron exhibiting both excitatory and inhibitory synaptic input from the BLA. (**D**) Distribution of CLA responses to photostimulation of BLA axon terminals. Top—Each square represents a single neuron (*n* = 100). Bottom—Fraction of EPSC responses detected in CLA projection neurons (*n* = 89) and interneurons (*n* = 11).

To measure the synaptic responses of CLA neurons (*n* = 100 from 14 mice) to BLA axon photostimulation, we recorded light-evoked postsynaptic currents at two different membrane potentials, −70 mV and −40 mV. At each holding potential, responses were recorded over 25 trials (Figure 3A,B; blue traces) and an average response was computed after excluding traces where no synaptic currents were evoked (Figure 3A,B; red traces). Holding at −70 mV maximized the amplitude of excitatory postsynaptic currents (EPSCs), which were inward at this potential and had a reversal potential near 0 mV. Holding at −70 mV also minimized the amplitude of inhibitory postsynaptic currents (IPSCs), which had a reversal potential near −70 mV under our experimental conditions. Thus, responses observed at −70 mV were primarily EPSCs (Figure 3A). In contrast, holding at a V_h_ of −40 mV increased the electrochemical driving force on chloride ions to reveal outward IPSCs (Figure 3B), while reducing the driving force on EPSCs to yield smaller inward EPSCs (Figure 3A). Thus, comparing responses measured at these two potentials allowed us to classify the inputs evoked by photostimulating BLA axons as excitatory, inhibitory, or mixed. To avoid spontaneous synaptic currents, only responses that were time-locked to the light flashes (between 5 and 25 ms after flash) were considered to be responses to BLA axon photostimulation. (Note that ChrimsonR has relatively slow activation kinetics that yield a latency of ~8 ms for evoking action potentials [53].) Light-evoked responses in CLA neurons were mostly excitatory (Figure 3A,D), with a small percentage of inhibitory responses (Figure 3B,D) and one case of dual EPSC and IPSC responses (Figure 3C,D).

Despite widespread expression of ChrimsonR in the BLA (e.g., Figure 1C), widefield photostimulation of BLA axons evoked postsynaptic responses in only half of the 100 CLA neurons examined (Figure 3D, top). This suggests that only a subset of CLA neurons receive BLA input. Both projection neurons and interneurons received excitatory synaptic input from the BLA (Figure 3D, bottom), and there were no significant differences in the EPSCs produced by BLA photostimulation in the two types of postsynaptic neurons (Figure S2). Although slice preparation inevitably severs some axons, widefield photostimulation is capable of evoking synaptic responses even from short axonal segments within the slice [39,54]; for this reason, most synaptic inputs present in vivo should be available for photostimulation, even if their axons were cut. Therefore, our observation of postsynaptic responses in only a fraction of CLA neurons is consistent with BLA axons targeting only a subset of CLA neurons.

#### 3.2.3. Variable, Unreliable and Small BLA Inputs to the CLA

**Figure 4:**
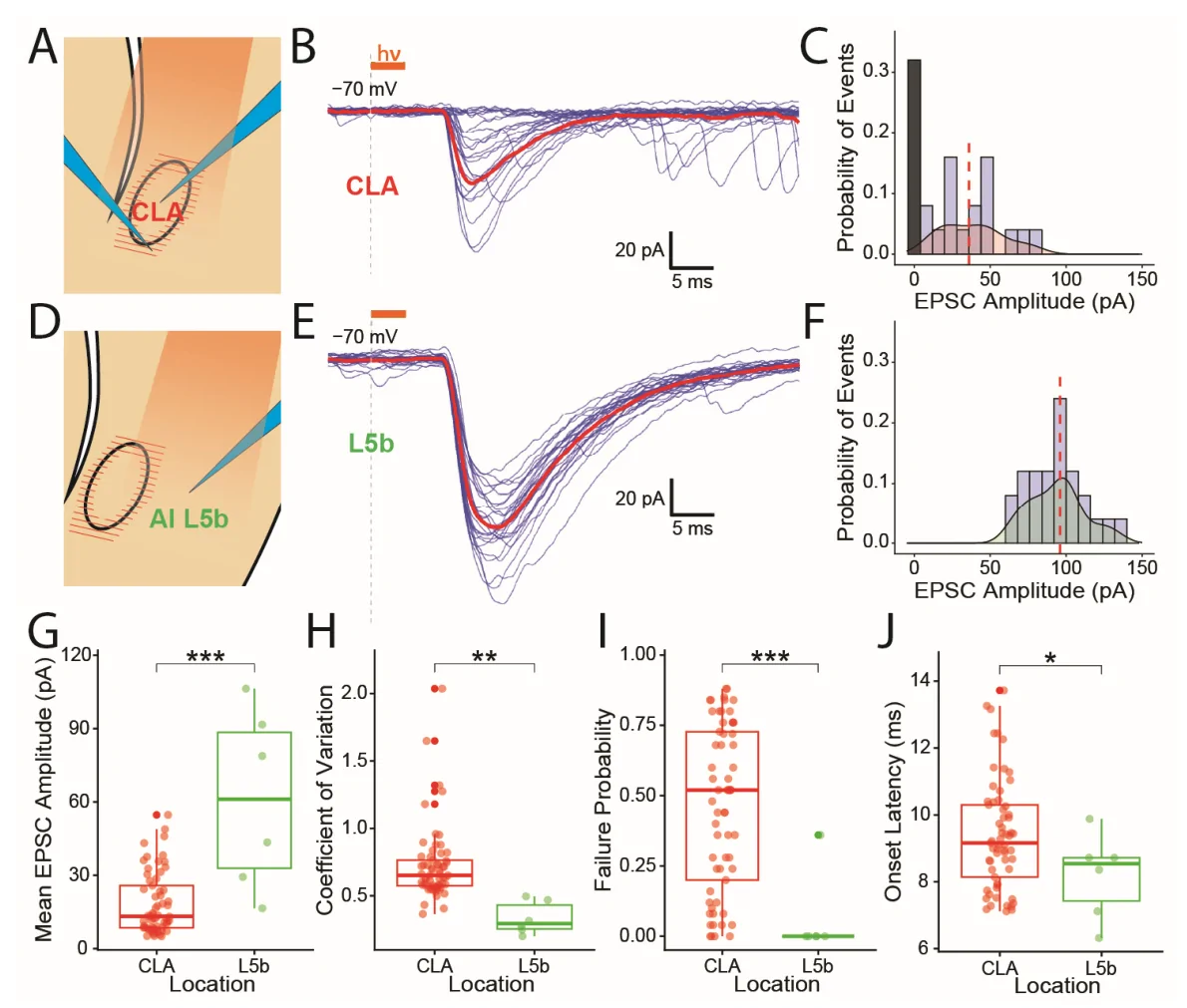
Comparison of excitatory BLA synaptic input to CLA and L5b AI neurons. (A,D) Neurons in CLA (**A**) and AI L5b (**D**) were patched to record their responses to photostimulation of BLA axons (red stippling) with orange light (shading). (**B**,**E**) Light-evoked EPSCs recorded in neurons in CLA (**B**) and AI L5b (**E**); both were held at −70 mV. Blue lines represent raw traces (25 trials) and bold red lines represent average EPSCs. Orange bar represents timing of photostimuli (4 ms duration) used to activate BLA axons. (**C**,**F**) Amplitude distributions (blue bars) for EPSCs shown in (**B**,**E**). Black bar represents response failures. Black line with the shaded area represents smoothed histogram using kernel density estimation. Red dotted line represents the mean EPSC values. (**G**–**J**) Comparison of properties of EPSCs recorded in CLA (red; *n* = 61 from 13 mice) and AI L5b neurons (green; *n* = 6 from five mice). (**G**) Mean amplitude of light-evoked EPSCs was significantly smaller in CLA neurons. Estimated difference between CLA and L5b neurons = −43.4 pA; 95% confidence interval = −56.4 to −30.4. (**H**) Light-evoked EPSCs were more variable in CLA neurons, illustrated by the high coefficient of variation. Estimated difference between CLA and L5b neurons = 0.39; 95% confidence interval = 0.16 to 0.62. (**I**) EPSC failure probability was much lower in L5b than in CLA. Estimated difference between CLA and L5b neurons = 0.47; 95% confidence interval = 0.25 to 0.70. (**J**) EPSC onset latency was slightly but significantly longer in CLA neurons compared to L5b. Estimated difference between CLA and L5b neurons = 1.57 ms; 95% confidence interval = 0.25 to 2.90. Significance testing used mixed-effects models with animals as the random effect to account for repeated sampling within animals; *p*-values were Benjamini–Hochberg-adjusted: *p.adj* < 0.05 for *, *p.adj* < 0.01 for **, and *p.adj* < 0.001 for ***.

We next characterized the properties of excitatory synaptic transmission between the BLA and the CLA (Figure 4A). Within individual CLA neurons (*n* = 61 from 13 mice), light-evoked EPSCs were highly variable in amplitude (Figure 4B,C). To obtain a reliable assessment of these responses in CLA neurons, BLA axons were photostimulated repeatedly; short-term synaptic plasticity was minimized by separating light flashes with relatively long inter-stimulus intervals (5 s) and this strategy was successful because we observed no systematic changes in EPSC amplitudes across the trials.

EPSC amplitudes fluctuated substantially from trial to trial (Figure 4B, blue traces) and spanned a broad range (Figure 4C, blue bars). Across all experiments, the mean amplitude of EPSCs was 18.4 pA (at −70 mV holding potential); this corresponds to a synaptic conductance of 0.26 nS and is several times smaller than the minimal current required to evoked APs in CLA projection neurons and interneurons [38]. To determine whether these EPSCs could evoke APs, in a subset of experiments we turned off the voltage clamp to record excitatory postsynaptic potentials (EPSPs) evoked by BLA photostimulation (see Figure 6A,B below). Photostimulation indeed evoked only subthreshold EPSPs that had a mean amplitude of 0.95 ± 0.26 mV (*n* = 16 cells from four mice). This indicates that the small amplitude and low conductance of the EPSCs evoked by BLA axon photostimulation renders them insufficient to generate APs in postsynaptic CLA neurons, even under conditions where many of these axons are being photostimulated.

Synaptic variability was quantified by the coefficient of variation (CV) of EPSCs, which was calculated as the standard deviation of EPSC amplitudes divided by the mean EPSC amplitude. Only trials with measurable EPSCs were used to compute the mean EPSC amplitude (Figure 4B, red line) and CV. The mean CV of CLA neuron EPSCs was 0.71 ± 0.25, indicating highly variable synaptic transmission. This is at, or slightly above, the high end of CV values calculated for other central excitatory synapses, which range from 0.2 to 0.7 [55,56]. Very similar results were obtained when using a different opsin, ChETA, to photostimulate BLA axons (Figure S5).

In some trials, photostimulation of BLA axons produced no detectable EPSCs (flat traces in Figure 4B), indicating synaptic failures (black bar in Figure 4C). To quantify synaptic reliability for each neuron, we measured failure probability which was defined as the fraction of trials without an EPSC. The mean failure probability at BLA-to-CLA synapses was 0.45 ± 0.29, indicating that even CLA neurons responding to BLA axon photostimulation produced EPSCs only about half the time. A similar result was observed when photostimulating BLA axons with ChETA (Figure S5) Thus, BLA inputs to CLA neurons are small, variable, and unreliable at both single-cell and population levels.

Our observation that the high EPSC failure rate was present whether photostimulating BLA axons with either ChrimsonR or ChETA suggests that the apparent unreliability of BLA–CLA synapses is not a consequence of inefficient photostimulation. To further consider this possibility, we also recorded EPSCs in neurons within the neighboring AI (Figure 2C,F) while using ChrimsonR to photostimulate the BLA axons that densely innervate the AI (Figure 2E,F) [57]. In contrast to CLA neurons, L5b neurons displayed larger, more consistent, and more reliable EPSCs (*n* = 6/8 responding neurons from seven mice). In the recordings from a L5b neuron shown in Figure 4E, median EPSC amplitude was 91.6 pA, which was higher than the median amplitude of 36.2 pA for the CLA neuron responses shown in Figure 4B. Even though the amplitudes of EPSCs in L5b neurons were larger, EPSC amplitudes still spanned a wide range (Figure 4F). Across all experiments, mean EPSC amplitude in L5b neurons was significantly (*p.adj* < 0.01) larger than in CLA neurons (Figure 4G). Calculation of the effect size yielded a Cohen’s d value of 1.70, indicating a large difference between EPSCs in the CLA and L5b. Thus, BLA input onto L5b AI neurons is relatively strong in comparison to CLA neurons. Importantly, there were no failures: EPSCs were observed in response to every photostimulus applied to the BLA axons. At the population level, both the CV (Figure 4H; Cohen’s d = 0.85, *p.adj* = 0.0022) and failure probability (Figure 4I; Cohen’s d = 1.10, *p.adj* = 0.00016) of CLA EPSCs were significantly higher than those recorded in L5b. The robustness of light-evoked EPSCs in L5b neurons confirms that our experimental conditions were adequate to photostimulate BLA axons within L5b. Thus, the weakness and unreliability observed when photostimulating BLA inputs to the CLA appear to be genuine features of this circuit.

Because polysynaptic pathways typically exhibit longer latencies than monosynaptic connections [58,59], we also measured the latency for EPSC onset. BLA-evoked EPSCs in the CLA showed a broad latency range, with a mean of 9.3 ± 1.6 ms. On average, the latency of EPSCs recorded in L5b neurons (8.2 ± 1.3 ms) was shorter than for CLA neurons (Figure 4J; Cohen’s d = 0.61, *p.adj* = 0.027). Accounting for the time required for ChrimsonR photostimulation to evoke action potentials (~8 ms) [53], the latencies of L5b neuron EPSCs are consistent with monosynaptic transmission. The broader range of EPSC latencies for CLA neurons raises the possibility that CLA responses may comprise a mix of both monosynaptic and polysynaptic components. We will address this issue in depth in Section 3.3.4.

### 3.3. Functional Connectivity Between the Basolateral Amygdala and the Claustrum Core and Shell

Given that the CLA is organized into core and shell domains, with the CLA shell apparently preferentially innervated by BLA fibers (Figure 2), we next asked whether this topography could explain our observation that only a subset of CLA neurons receive BLA input (Figure 3C). We used two criteria to divide the CLA into core and shell compartments: regions of dense BLA fiber projections were used to define the shell (Figure 2E), while post hoc PV immunocytochemistry was used to delineate the PV-rich core (Figure 2D). Neurobiotin filling (Figure 2C) was used to identify the positions of CLA neurons relative to the core boundary (Figure 2F) and thereby define whether these neurons were in the core or shell.

#### 3.3.1. BLA Input Is Similar in the CLA Core and Shell

**Figure 5:**
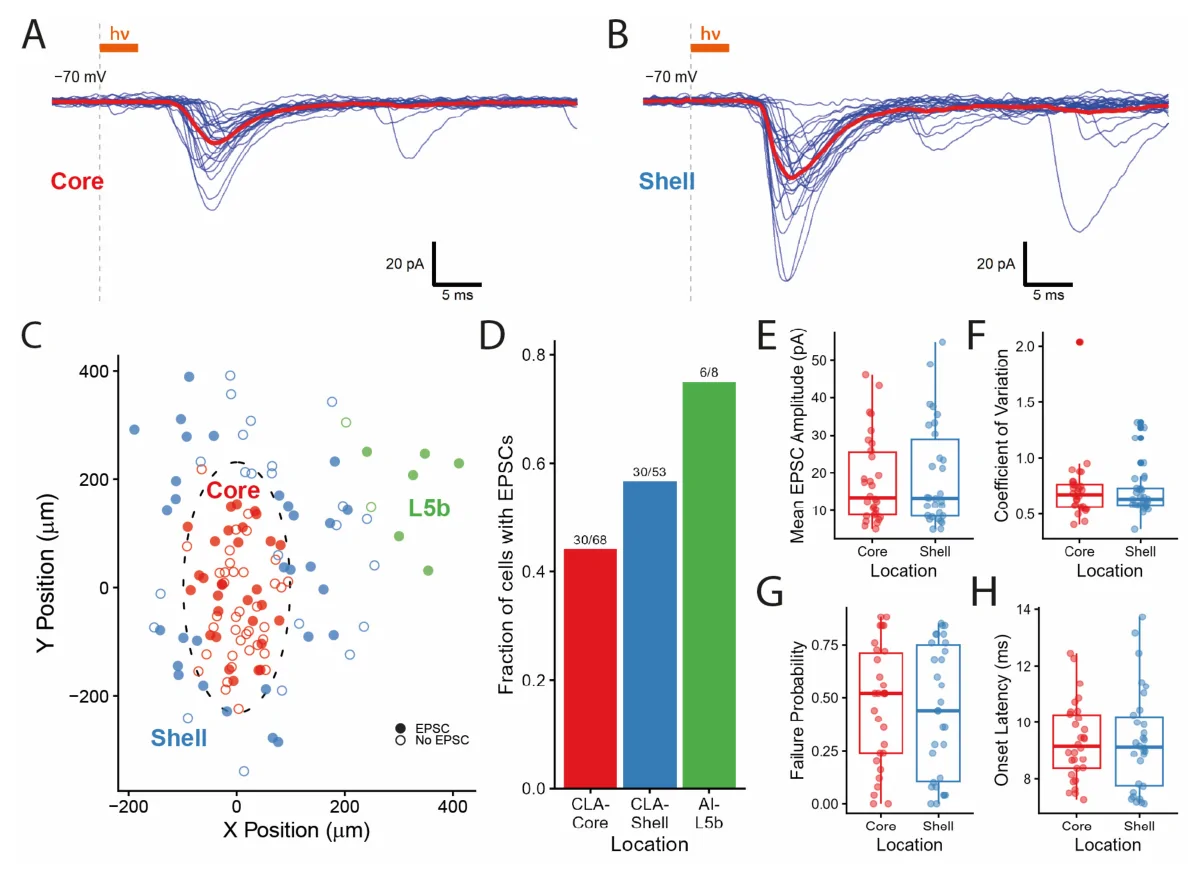
Comparison of EPSCs recorded in CLA core and shell neurons. (**A**,**B**) Representative light-induced EPSCs recorded in neurons held at −70 mV during photostimulation of BLA axons. Blue lines represent raw traces (25 sweeps) and bold red lines represent average EPSCs. Orange bars represent timing of photostimuli (4 ms duration) used to activate BLA axons. (**A**) EPSCs recorded in a CLA core neuron. (**B**) EPSCs recorded in a CLA shell neuron. (**C**) Location of neurons that did (filled symbols) or did not (open symbols) respond to photostimulation of BLA axons: neurons were in CLA core (red), CLA shell (blue), and AI L5b (green). Dashed line depicts average CLA core dimensions across experiments. (**D**) The fraction of cells with detectable EPSCs was not significantly different between CLA core and shell neurons (odds ratio = 0.68, 95% confidence interval = 0.30 to 1.53, generalized linear mixed-effects model), between CLA core and AI L5b neurons (odds ratio = 0.28, 95% confidence interval = 0.05 to 1.57) or between CLA shell and L5b neurons (odds ratio = 0.23, 95% confidence interval = 0.03 to 1.95). (**E**–**H**) Light-induced EPSCs in both CLA core (red) and shell (blue) neurons had similar properties; estimates for core−shell differences in: (**E**) mean amplitudes (estimated difference = −0.67 pA; 95% confidence interval = −6.34 to 5.01); (**F**) coefficient of variation (estimated difference = 0.05; 95% confidence interval = −0.09 to 0.19); (**G**) failure probabilities (estimated difference = 0.002; 95% confidence interval = −0.13 to 0.14); and (**H**) onset latency (estimated difference = −0.10 ms; 95% confidence interval = −0.89 to 0.70). Differences were non-significant in all EPSC response properties (all *p.adj* ≥ 0.05, mixed-effects models).

We recorded from neurons in the CLA core (*n* = 68 from 14 mice) and shell (*n* = 53 from 15 mice) to compare their responses to BLA axon photostimulation. Such photostimulation evoked EPSCs in neurons in both locations (Figure 5A,B). The spatial distribution of core and shell neurons that responded to photostimulation of BLA axons (Figure 5C, filled symbols) revealed that these cells were intermingled with neurons that did not respond (Figure 5C, open symbols). Quantitatively, the fraction of cells exhibiting light-evoked EPSCs was below 60% in both the core and shell regions (Figure 5D) and was not significantly different (odds ratio = 0.68, *p.adj* = 0.35). Thus, despite a clear anatomical difference in BLA axon density that we observed between the CLA core and shell (Figure 1 and Figure 2), the fraction of CLA neurons located in these two compartments that responded to BLA axon photostimulation were not different (Figure 5C). To determine whether this relatively low connection probability was specific to the CLA, we also compared the fraction of L5b cells within the AI that exhibited EPSCs. The response probability of these neurons was not significantly different (Figure 5D; *p.adj* = 0.27) when compared to either CLA core neurons (odds ratio = 0.28) or to CLA shell neurons (odds ratio = 0.23).

#### 3.3.2. EPSCs from BLA Input Are Uniform Across the CLA Core and Shell

Despite BLA connection probability being similar for neurons in the CLA core and shell, we next asked whether EPSC properties differed between the two regions. In fact, EPSCs were remarkably similar in both regions, with effect sizes for differences between all properties being negligible to small (*n* = 31 core cells from 12 mice and 30 shell cells from 10 mice). Mean EPSC amplitudes did not differ between core and shell neurons, indicating comparable synaptic strength in both regions (Figure 5E; Cohen’s d = −0.06, *p.adj* = 0.95). Trial-to-trial variability in EPSC amplitude was prominent in both regions (Figure 5A,B) and yielded nearly identical CV values for core and shell EPSCs (Figure 5F; Cohen’s d = 0.19, *p.adj* = 0.82). Mean failure probabilities also were similar in both regions (Cohen’s d = 0.01, *p.adj* = 0.97) and spanned similar ranges (SDs of 0.28 and 0.31), indicating equivalent synaptic reliability (Figure 5G). Finally, EPSC onset latencies were comparable between core and shell neurons (Figure 5H; Cohen’s d = −0.07, *p.adj* = 0.95). All these results are consistent with CLA core and shell neurons having similar underlying circuit architecture.

#### 3.3.3. Short-Term Synaptic Plasticity at the BLA-to-CLA Synapse

**Figure 6:**
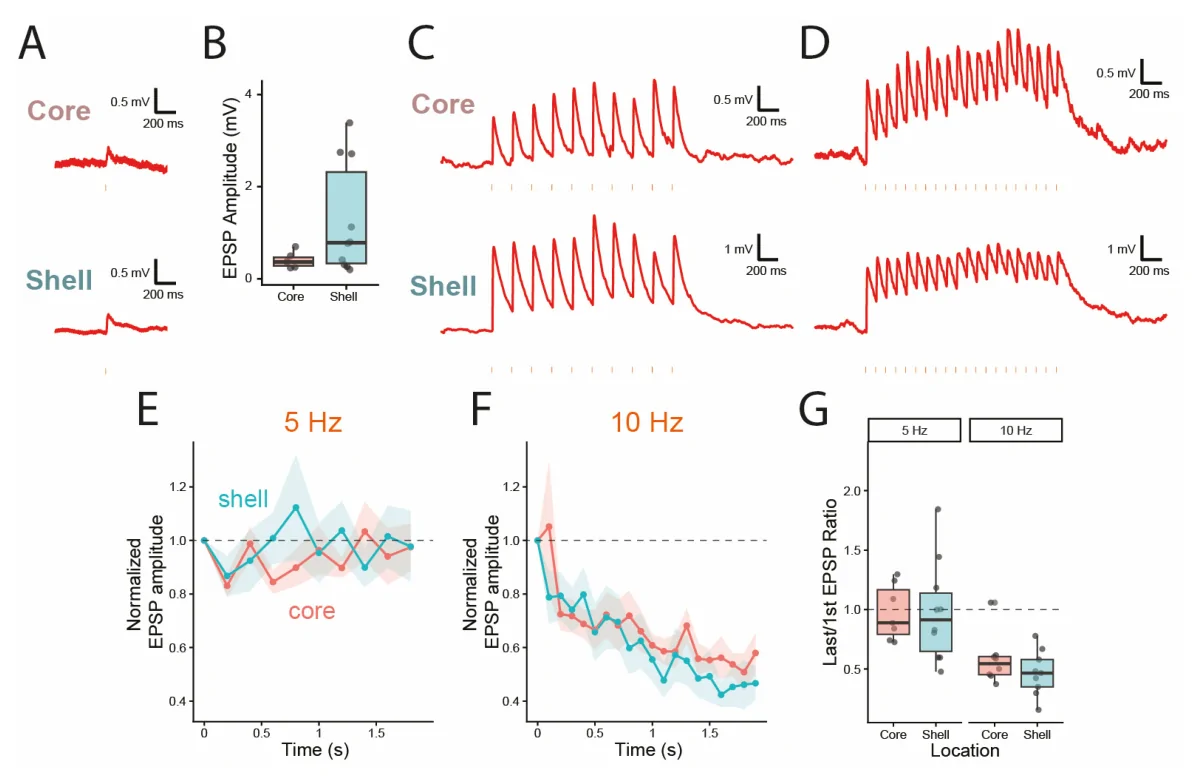
Short-term synaptic plasticity of BLA-to-CLA circuits. (**A**) Photostimulation of BLA axons evoked subthreshold excitatory postsynaptic potentials (EPSPs) in CLA core and shell neurons. (**A**,**C**,**D**) EPSP responses (red lines; means of 20 sweeps) evoked by photostimuli (4 ms duration; orange bars at bottom) of BLA axons. (**B**) EPSPs in CLA core and shell have similarly small amplitudes. (**C**,**D**) EPSPs evoked in CLA core and shell neurons by repetitive photostimulation of BLA axons at 5 Hz (**C**) and 10 Hz (**D**). (**E**,**F**) Normalized amplitudes of EPSPs evoked in core (vermilion) and shell (cyan) neurons during 5 Hz (**E**) and 10 Hz (**F**) photostimulation. Bold lines represent mean values and shaded zones represent ±1 standard error of the means. (**G**) Short-term synaptic plasticity represented as the ratio of amplitudes of last/first EPSPs at 5 Hz (*n* = 7 core neurons from four mice, 10 shell neurons from three mice) and 10 Hz (*n* = 8 core neurons from four mice, 9 shell neurons from three mice). Core and shell neurons showed similar synaptic depression at 10 Hz: estimated difference = 0.11; 95% confidence interval = −0.11 to 0.33. Significance testing used mixed-effects models (animal as random effect); *p.adj* = 0.70 (Benjamini–Hochberg-corrected). Dashed line indicates no difference between amplitudes of first and last EPSPs.

We next asked whether the BLA-to-CLA circuit exhibits short-term synaptic plasticity in the core or shell neurons by measuring EPSPs evoked by photostimulation of BLA axons. Such stimuli evoked only subthreshold EPSPs in both CLA core neurons (Figure 6A, top; *n* = 6 from four mice) and shell neurons (Figure 6A, bottom; *n* = 10 from three mice). EPSP amplitude was similar between core and shell neurons (Figure 6B), consistent with our measurements of EPSCs in these neurons (Figure 5E). We examined short-term plasticity by measuring the amplitude of EPSPs evoked by repetitive photostimulation of BLA axons. EPSPs measured during 5 Hz photostimulation (Figure 6C) were constant in amplitude over the duration of 2 s long trains (Figure 6E,G). This was the case in both core and shell neurons (Figure 6C,E,G) and indicates minimal synaptic plasticity at this rate of synaptic activity. During 10 Hz photostimulation, there was pronounced temporal summation of individual EPSPs (Figure 6D) but a net depression of EPSP amplitude over the course of 2 s (Figure 6D,F). The amount of synaptic depression was not significantly different between the claustral core and shell neurons (Figure 6G; Cohen’s d = 0.58, *p.adj* = 0.70).

In summary, basal synaptic transmission between presynaptic BLA axons and postsynaptic CLA neurons does not differ between the CLA core and shell. Further, synaptic depression is evident at 10 Hz and this form of activity-dependent synaptic plasticity also does not differ between core and shell neurons.

#### 3.3.4. A Monosynaptic BLA-to-CLA Circuit

The presence of functional BLA synapses in the CLA core, despite the low axon density there, raises the question of whether BLA inputs directly terminate onto CLA core neurons. We next addressed this question by asking whether BLA inputs to CLA neurons are monosynaptic. For this purpose, we used a pharmacological strategy developed by Petreanu et al. [39] that is based on treatment with tetrodotoxin (TTX) and 4-aminopyridine (4AP) [39,40,48]. TTX blocks voltage-gated sodium channels [60], abolishing action potential propagation in BLA axons and thereby eliminating all postsynaptic responses. Subsequent addition of 4AP, a blocker of voltage-gated potassium channels, promotes local calcium spikes at axon terminals [61] and can restore transmitter release from directly photostimulated terminals [39]. Thus, EPSCs observed in the presence of both drugs (TTX+4AP) should reflect direct, monosynaptic connections because action potentials in intermediate neurons of any polysynaptic circuit would still be blocked.

**Figure 7:**
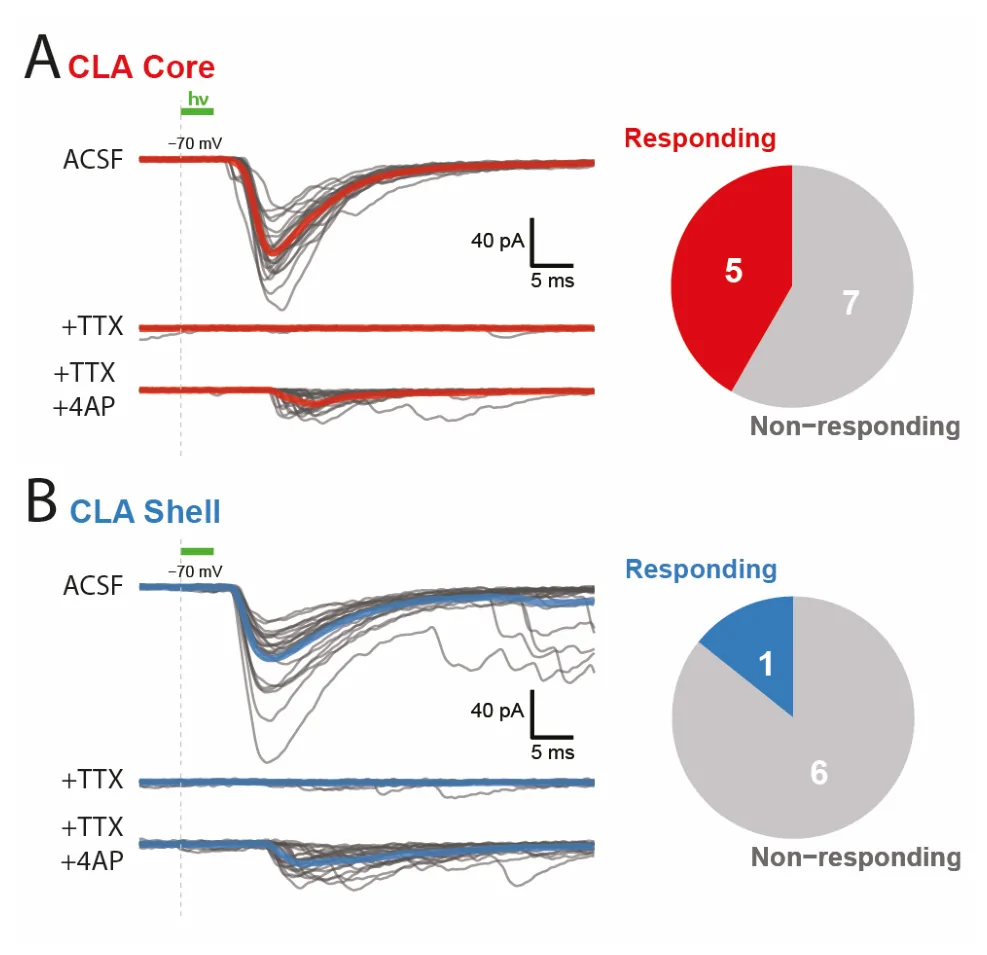
Monosynaptic BLA inputs to both CLA core (**A**) and shell (**B**). Left: EPSCs recordings; gray lines represent raw traces and bold lines represent average EPSCs. Neurons were held at −70 mV. Green bars indicate timing of photostimuli (4 ms duration) applied to ChETA-expressing BLA axons. Right: Fraction of neurons that did (Responding) or did not (Non-responding) produce EPSCs in TTX+4AP conditions.

For neurons in both the CLA core (Figure 7A) and the shell (Figure 7B), light-evoked EPSCs were sequentially recorded in control conditions, in the presence of TTX and then in the presence of TTX+4AP. As expected, EPSCs observed in control conditions (Figure 7A,B—top traces) were abolished by TTX (Figure 7A,B—middle traces). In TTX+4AP conditions, five out of 12 CLA core neurons exhibited EPSCs (Figure 7A—lower traces and pie chart). This demonstrates the existence of direct monosynaptic connections between the BLA and neurons within the CLA core, despite the low density of BLA fibers in the core. Similarly, one out of seven neurons in the CLA shell exhibited EPSCs in the presence of TTX+4AP (Figure 7B—lower traces and pie chart), indicating the existence of direct monosynaptic BLA-to-CLA shell neuron connections. Thus, at least a subset of the excitatory connections from the BLA to the CLA core and the CLA shell are clearly monosynaptic, although other inputs may include polysynaptic components.

### 3.4. Basolateral Amygdala Connectivity with Claustral Projection Neuron Subpopulations

CLA neurons projecting to specific cortical targets are partially segregated into zones and inputs from related cortical areas preferentially innervate the same zones [4,9,31,62,63,64]. For example, CLA neurons projecting to M1 and RSC occupy distinct, minimally overlapping territories: the former reside within the dorsal CLA (dCLA), while the latter are within the CLA itself [31]. In addition, some inputs to the CLA selectively enrich the CLA shell but not the PV-rich CLA core (see Figure 1 and Figure 2, as well as references [9,12,49]). These findings have led to the idea that different CLA zones form partially segregated input–output modules [40,65].

We determined whether such cellular topology reflects CLA functional organization by examining BLA inputs onto CLA neurons projecting to M1 or to RSC. The clear topographical segregation of M1-projecting and RSC-projecting CLA neurons [31] makes these projection-defined cell populations useful for determining whether functional input to the CLA parallels cellular zoning. We first revisit the anatomical relationship between CLA neurons projecting to M1 or RSC and then will consider BLA innervation of each projection neuron subpopulation.

#### 3.4.1. Topological Distribution of CLA Projection Neurons

**Figure 8:**
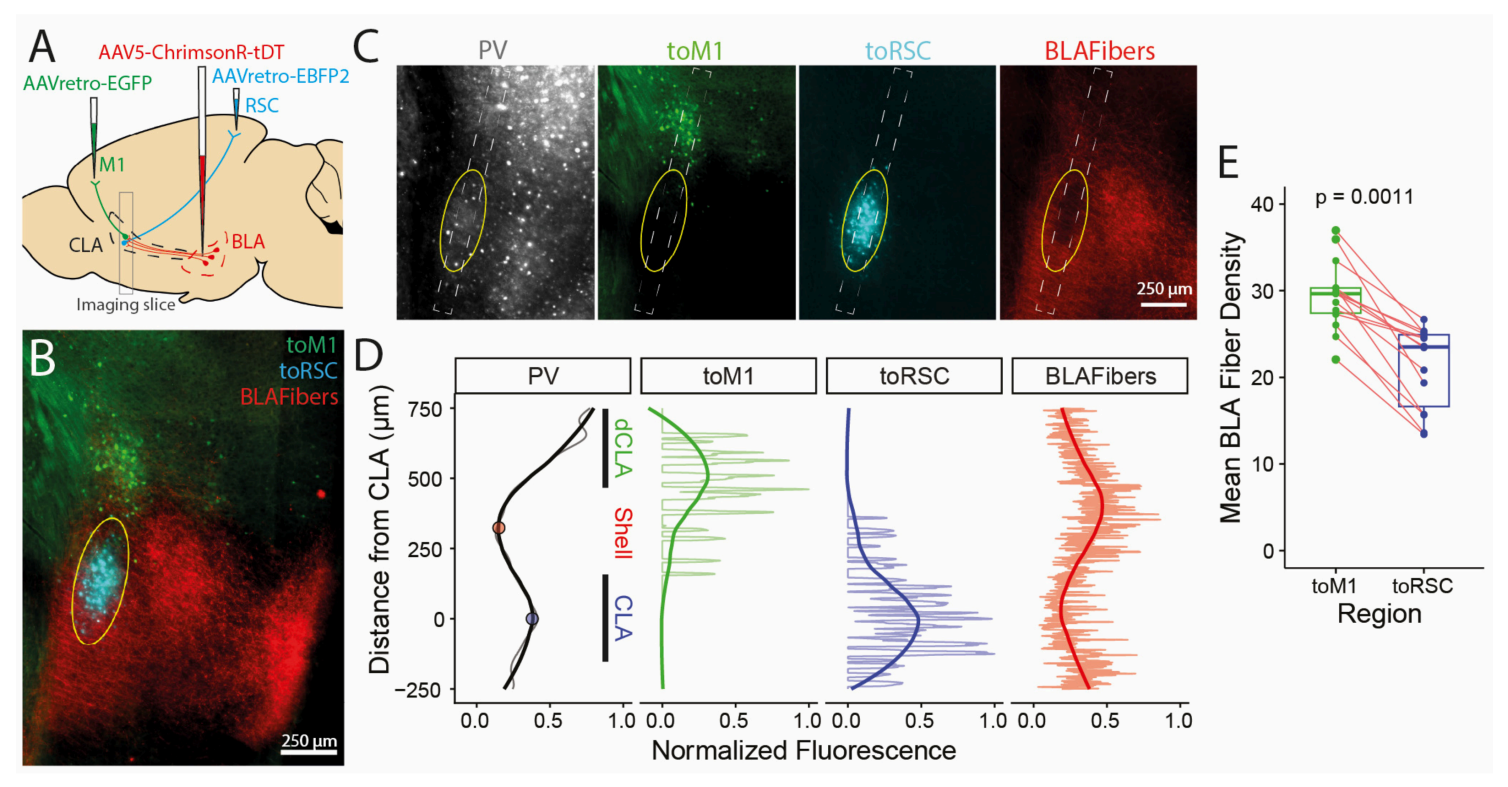
Locations of M1-projecting and RSC-projecting CLA neurons and their BLA input. (**A**) Schematic illustrating viral injection sites and resulting tracer expression in the CLA. Retrograde tracers labeled M1-projecting CLA neurons with EGFP (green) and RSC-projecting CLA neurons with EBFP2 (blue). BLA axons were labeled with tdTomato (tDT) using an anterograde AAV (red). (**B**) Composite fluorescence image of coronal section of CLA labeled as indicated in (**A**). (**C**) Magnified images of separate fluorescence channels shown in (**B**). White dashed lines indicate region used for profile plots in (**D**). Yellow ellipses in (**B**,**C**) represent the CLA core, as delineated by PV staining (C, left-most panel). (**D**) Quantified profile plots from images in (**C**), showing the distribution of normalized fluorescence densities along the longitudinal axis of the CLA. Bold lines represent average fits using local polynomial regression (LOESS). Raw values are displayed in thinner background lines. PV staining (black) was used to identify CLA and CLA shell/dCLA intersection. Colored circles and vertical lines represent region assignments based on PV expression—CLA and vCLA shell/dCLA. (**E**) Mean BLA fiber density in regions within *toM1* (green) and *toRSC* (blue). Paired regions are linked by red lines (*n* = 2 mice, 14 sections). Wilcoxon signed-rank test was used to test statistical significance.

To label CLA neurons that project to M1 or to RSC, retrograde AAVs expressing different fluorophores were injected into each of these areas (Figure 8A). Neurons projecting to M1 (*toM1*) and neurons projecting to RSC (*toRSC*) were present in clearly distinct CLA zones (Figure 8B), as previously reported [31]. PV immunohistochemistry again was used to identify the CLA core as a landmark (PV in Figure 8C) and revealed that *toM1* neurons clustered in the dCLA, dorsal to the CLA core (Figure 8C). In contrast, *toRSC* neurons clustered within the CLA core (Figure 8C). These relationships were quantified via masked spatial profiles (Figure 8D and Figure S4A).

Given the ill-defined boundaries between CLA subregions, we measured fluorescence intensities across the full dorsal–ventral extent of the dCLA and CLA (Figure 8C and Figure S4A). Raw fluorescence values were smoothed to yield average density traces (Figure 8D). The first PV peak was defined as CLA core and the subsequent trough in PV fluorescence as the transition between the CLA shell and dCLA (Figure 8D, PV). Comparing the distributions of M1-projecting and RSC-projecting neurons along this axis, *toRSC* somata were clearly confined to the CLA core, as indicated by the near-complete overlap between peaks of *toRSC* and PV labeling (Figure 8D, PV and *toRSC*). In contrast, *toM1* neurons clustered in the dCLA, approximately 500 µm dorsal to the PV-rich CLA core (Figure 8D). These results provide quantitative support for the conclusion that M1-projecting and RSC-projecting CLA neurons occupy distinct, projection-based zones within the CLA complex. This zone-specific localization was consistent along the rostral–caudal axis (Figure S4B–D): *toM1* neurons clustered in dCLA and rarely appeared in CLA (Figure S4B), whereas *toRSC* neurons occupied CLA core and were rarely observed in dCLA (Figure S4C). Thus, M1- and RSC-projecting CLA neurons form largely non-overlapping topological subpopulations throughout the entire claustrum complex.

#### 3.4.2. BLA Axons Overlap More with M1-Projecting CLA Neurons Compared to RSC-Projecting Neurons

To compare the BLA input received by these two populations of CLA projection neurons, BLA axons again were labeled with an anterograde AAV expressing red-tagged ChrimsonR that was injected into the BLA. The red-labeled BLA axons (*BLAFibers*) were enriched in the region surrounding—albeit minimally within—the central region of the CLA core (Figure 8B,C), consistent with the results shown in Figure 1E and Figure 2D. BLA axon density was quantified across the dorsal–ventral CLA axis (Figure 8C and Figure S4D, *BLAFibers*). A comparison of the distributions of *toM1* and *toRSC* somata with that of *BLAFibers* revealed an inverse relationship between the density of *toRSC* somata and *BLAFibers* (Figure 8D). This suggests that *toRSC* neurons in the CLA core might receive little BLA input. Conversely, *BLAFibers* density was relatively high near the *toM1* peak (Figure 8D), suggesting that BLA input onto *toM1* neurons could be substantial.

These anatomical relationships were consistent along the rostral–caudal axis: *BLAFibers* maintained peak densities flanking the CLA core and remained sparse within it (Figure S4A–D). *BLAFibers* distribution was consistently the inverse of that of *toRSC* somata (Figure S4C,D), again suggesting minimal BLA input to many *toRSC* neurons. In contrast, *BLAFibers* overlapped more strongly with *toM1* somata in rostral sections (Figure S4B,D, top row), consistent with stronger BLA input to *toM1* neurons rostrally. In intermediate and caudal sections, *BLAFibers* appeared reduced near the *toM1* peak (Figure S4B,D, middle and bottom rows), suggesting weaker BLA innervation of *toM1* neurons posteriorly. This may partly reflect limitations of profile averaging, where weaker *BLAFibers* signals in intermediate/caudal sections could cause a slight misalignment of peaks.

To more accurately compare BLA innervation of M1-projecting and RSC-projecting CLA neurons, we measured the fluorescence of *BLAFibers* in 50 µm × 100 µm areas centered on the peaks of *toM1* or *toRSC* soma density. Mean *BLAFibers* fluorescence intensity within these regions was compared across sections and animals (Figure 8E). This comparison was based on samples from two mice (14 sections total); because the unit of biological replication for this analysis is the animal, rather than the individual section, this result should be interpreted with some caution. However, for both mice, *BLAFibers* fluorescence was consistently higher in the location of *toM1* neurons than the location of *toRSC* neurons in the same section and was also consistent across the rostral–caudal axis (Figure S4B–E), indicating stronger anatomical overlap between BLA axons and M1-projecting CLA neurons. Together, our tracing data suggest that BLA input varies systematically with projection target and CLA topology and that BLA innervation may be heavier for M1-projecting than for RSC-projecting CLA neurons.

#### 3.4.3. BLA Preferentially Innervates RSC-Projecting CLA Neurons Compared to M1-Projecting Neurons

**Figure 9:**
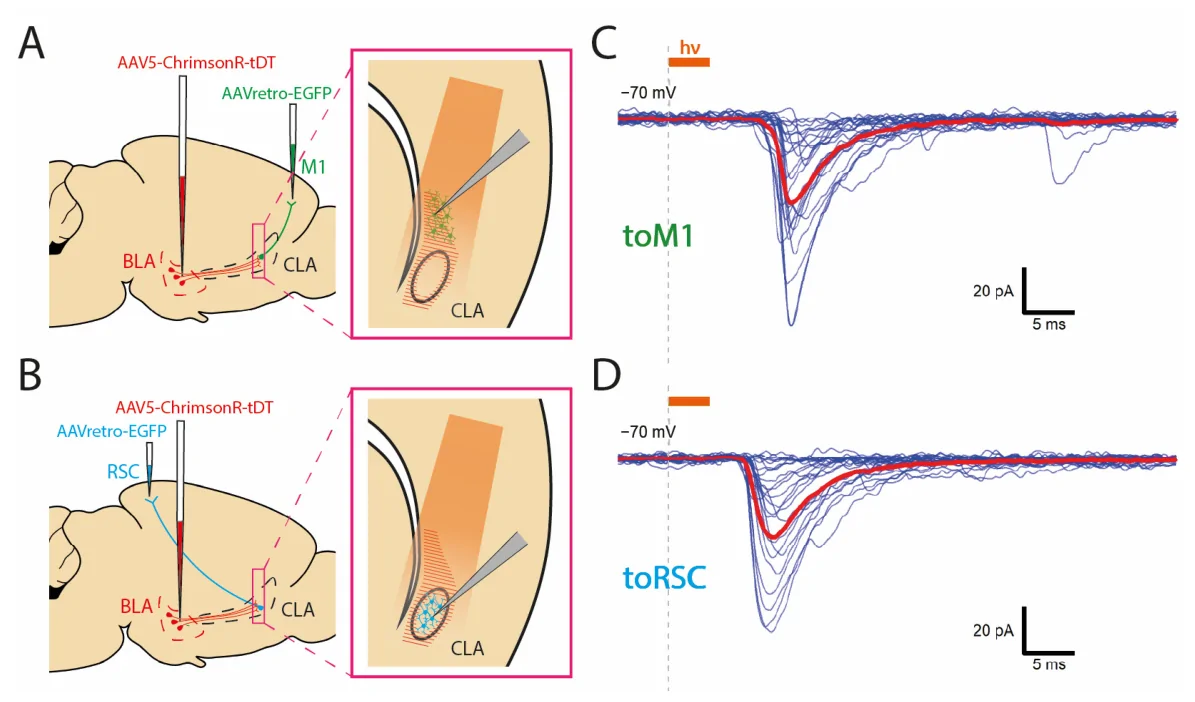
Comparison of BLA excitation of CLA neurons projecting to M1 and RSC. (**A**,**B**) Schematic of experimental strategy. Left: Injected AAVs expressed ChrimsonR in BLA axons and labeled either M1-projecting CLA neurons (**A**) or RSC-projecting CLA neurons (**B**) Right: Whole-cell patch clamp recordings from labeled neurons in response to photostimulation (orange shaded area) of BLA axons. Black ellipse represents CLA boundary; hatched red areas represent *BLAFibers*. (**C**,**D**) EPSCs recorded in (**C**) labeled M1-projecting CLA neurons (*toM1*) and (**D**) RSC-projecting CLA neurons (*toRSC*) in response to photostimulation (orange bars) of BLA axons. Blue lines represent raw traces (25 trials) and bold red lines represent average responses. EPSCs were recorded at −70 mV holding potential.

We next determined whether these anatomical differences in BLA axons reflect differences in BLA synaptic input by measuring CLA projection neuron responses to BLA axon photostimulation. ChrimsonR was again expressed in BLA neurons using an anterograde AAV to enable photostimulation of BLA axon terminals (Figure 9A,B—left column). CLA neurons projecting to M1 (Figure 9A) or to RSC (Figure 9B) were retrogradely labeled, allowing visually targeted whole-cell recordings from projection-defined neurons (Figure 9A,B—left). By recording synaptic currents in labeled neurons during photo -stimulation of BLA axons (Figure 9A,B—right), we were able to directly compare BLA synaptic input onto CLA neurons projecting to M1 (*n* = 27 from seven mice) versus those projecting to RSC (*n* = 39 from six mice).

Photostimulation of BLA axons evoked primarily excitatory postsynaptic currents (EPSCs) in both M1-projecting and RSC-projecting claustral neurons (Figure 9C,D). In a small number of experiments, BLA photostimulation also evoked IPSCs in *toM1* neurons (one out of 24) and in *toRSC* neurons (two out of 30), indicating that BLA inputs on both types of projection neuron are predominantly excitatory. The presence of robust inward EPSCs following BLA photostimulation indicates that BLA inputs can excite both M1-projecting and RSC-projecting CLA neurons.

**Figure 10:**
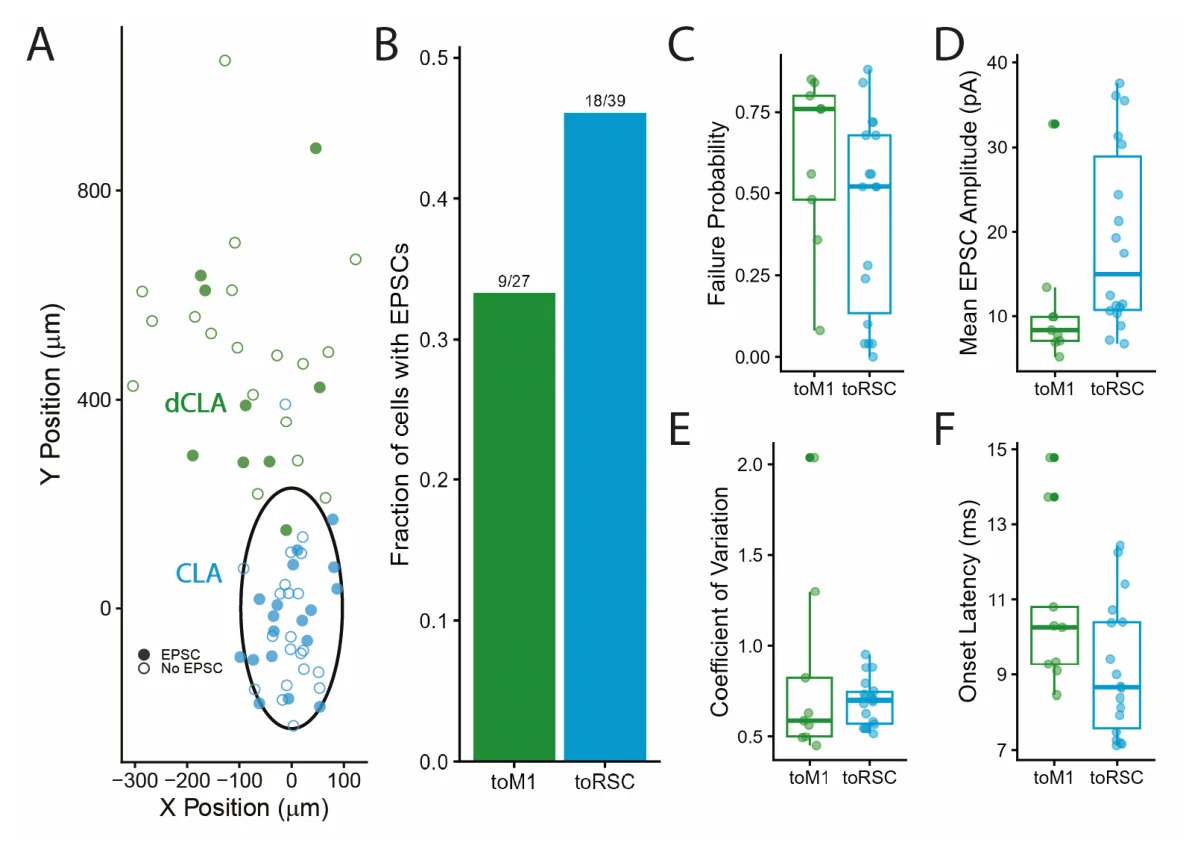
Comparison of EPSCs produced in RSC-projecting and M1-projecting CLA neurons in response to BLA axon photostimulation. (**A**) Distribution of M1-projecting CLA neurons (*toM1*, green) and RSC-projecting CLA neurons (*toRSC*, blue) responding (filled symbols) or not responding (open symbols) to BLA axon photostimulation. (**B**) Fraction of cells exhibiting EPSC responses to BLA axon photostimulation did not differ significantly (odds ratio = 0.57, 95% confidence interval = 0.15 to 2.14) for *toRSC* (blue) neurons and *toM1* neurons (green). (**C**–**F**) EPSC response properties were similar between both neuron types in regard to their EPSC; estimates for toM1–toRSC differences in: (**C**) failure rate (estimated difference = 0.09; 95% confidence interval = −0.25 to 0.44); (**D**) mean amplitude (estimated difference = −6.57 pA; 95% confidence interval = −18.2 to 5.03); (**E**) coefficient of variation (estimated difference = 0.13; 95% confidence interval = −0.18 to 0.44); and (**F**) onset latency (estimated difference = 1.26 ms; 95% confidence interval = −1.24 to 3.76). All EPSC properties were not significantly different between *toM1* and *toRSC* neurons (*p.adj* ≥ 0.05, mixed-effects models).

To determine whether BLA input probability differed between M1-projecting and RSC-projecting CLA neurons, EPSC responses (excluding failures) were averaged to generate a mean EPSC trace for each individual neuron (red lines in Figure 9C,D). These average responses were then used to compare EPSC properties between the two projection-defined neuron populations. The spatial distribution of CLA neurons that did or did not produce EPSCs in response to BLA photostimulation are shown in Figure 10A. In contrast to expectations based on the anatomical observations described above (Figure 8), light-evoked EPSCs were observed in both RSC-projecting neurons within the CLA core and in M1-projecting neurons in the dCLA (Figure 10A). Within each zone, responding neurons did not form obvious finer-scale clusters and, instead, were scattered across the dCLA for *toM1* and across the CLA core for *toRSC* (Figure 10A). We quantified EPSC response probability for each neuron type. Of the 27 *toM1* neurons examined, nine produced EPSCs in response to BLA axon photostimulation (Figure 10B). In comparison, 18 out of 39 toRSC neurons were responsive (Figure 10B), although this difference was not statistically significant (odds ratio = 0.57, *p.adj* = 0.41). The fact that both projection neuron populations had a relatively low response probability further suggests that BLA axons innervate only a subset of each neuronal population.

#### 3.4.4. Similar BLA Connections for RSC-Projecting and M1-Projecting CLA Neurons

Finally, we asked whether the properties of BLA synaptic input differed between *toM1* (*n* = 9 from six mice) and *toRSC* (*n* = 18 from five mice) projection neurons. We first compared EPSC failure probability and found that BLA axon photostimulation produced EPSCs in fewer than half of trials for both types of projection neuron (Figure 10C). Failure probabilities were statistically indistinguishable between CLA neurons projecting to M1 and those projecting to RSC (Cohen’s d = 0.43, *p.adj* = 0.62). However, *toRSC* neurons exhibited a broader range of failure probabilities, including 4 neurons that had highly reliable responses (failure probability < 0.10 in Figure 10C). This hints at the existence of a subpopulation of *toRSC* neurons with particularly reliable BLA synapses.

While mean EPSC amplitude appeared different between *toRSC* and *toM1* neurons (Figure 10D), with a moderate/large effect size (Cohen’s d = −0.90), there was no statistically significant difference between the two groups (*p.adj* = 0.62). EPSC amplitudes in *toRSC* neurons did span a wider range, suggesting greater heterogeneity in connection strength onto *toRSC* neurons relative to *toM1* neurons (Figure 10D). The mean CVs for EPSCs did not differ significantly (Cohen’s d = 0.66, *p.adj* = 0.62; between *toM1* and *toRSC* neurons, indicating similar trial-to-trial variability of BLA transmission in both populations (Figure 10E). Finally, EPSC onset latencies were comparable (Figure 10F; Cohen’s d = 0.79, *p.adj* = 0.62) for the two projection neuron types, consistent with similar underlying BLA input circuits for both populations.

In summary, our analyses demonstrate that BLA inputs to both M1-projecting and RSC-projecting CLA neurons are predominantly excitatory. Despite a higher BLA fiber density near M1-projecting neurons, the overall transmission properties of both synapses are similar. Thus, even though BLA fiber density is anatomically quite different for these two projection neuron populations, the functional input they receive is comparable.

## 4. Discussion

Our results define how BLA inputs are anatomically organized within the CLA and how they functionally engage distinct subpopulations of claustral neurons. We found that BLA innervation of the CLA is shaped in a ring-like structure within the shell and surrounds the PV-rich core. Functionally, BLA input provides small and variable excitatory input onto a subset of neurons distributed across both the CLA core and shell. We further demonstrate that, despite greater anatomical overlap between BLA fibers and M1-projecting CLA neurons, BLA excitation is similar for RSC-projecting neurons, revealing a second dissociation between axon density and functional drive. These findings suggest a model in which BLA input contributes a weak, sparse, and relatively uniform excitatory bias to CLA activity. We note that our experiments were conducted exclusively in male mice. Because sex differences have been reported for amygdala connectivity [66] and in CLA neuron function [67] in mice, it remains to be determined whether the anatomical and functional organization of the BLA–CLA pathway described here is the same in females.

### 4.1. Core–Shell Segregation of BLA–CLA Anatomical Connectivity

The borders and cellular organization of the claustrum have been debated for decades. Accumulating genetic and anatomical data increasingly support the idea that the CLA is subdivided into at least two zones: the PV-rich core and a surrounding shell [49,63,65,68]. Our viral tracing reveals an unexpected topological specificity in BLA–CLA connectivity that strongly reinforces this view: we found that both BLA-projecting CLA neurons and BLA axons are enriched in the shell region and sparse in the CLA core.

Our key to clarifying this organization relied on the use of a tracing strategy that was BLA-focused. This contrasts with earlier lower-resolution studies that employed viral tracing methods based on CLA-focused strategies and inferred bidirectional connectivity between the BLA and the CLA core [13,31]. In the CLA-to-BLA direction, labeling of apparently “core-specific” CLA neurons suggested that core neurons project to BLA. This conclusion appears to be incorrect and probably arose because there was no attempt to identify the spatial relationship between the neurons that project to the BLA and the CLA core; we have sidestepped this problem by simultaneously labeling the core via PV staining while retrogradely labeling BLA-projecting neurons of the CLA. In the BLA-to-CLA direction, previous transsynaptic labeling from the CLA core similarly suggested BLA inputs to only core neurons. While we have also detected functional connectivity between the BLA and core neurons, this probably results from BLA axons present in the CLA shell innervating the distal dendrites of core neurons. These axons in the CLA shell also provide similar input to neurons whose cell bodies reside within the shell. Thus, BLA inputs in the shell contact neurons that have cell bodies located in both the shell and the core.

Our finding of bidirectional BLA–CLA connectivity that is anatomically confined to the shell provides a further indication that the CLA has a shell region distinct from the core in both its input and output connectivity (although this distinction is less apparent in synaptic function). Importantly, this topological segregation is not unique to BLA–CLA circuitry. Other studies have reported that the orbitofrontal and auditory cortices also preferentially innervate the shell, whereas the visual and anterior cingulate cortices target the core [49]. Interneuron distributions also differ between core and shell, with distinct core enrichment of PV versus VIP/SST subtypes [38,66]. Together with our data, these findings argue that the CLA is partitioned into at least two partially segregated modules—core and shell—with distinct combinations of inputs, outputs, and local circuitry.

### 4.2. BLA Forms Weak, Variable Synapses on Only a Subset of CLA Neurons

Our current anatomical findings, along with previous studies [8,15,31], suggest a direct BLA-to-CLA pathway that could serve as a conduit to deliver valence-related and emotion-related information to the CLA. However, the function of this pathway had not been examined previously. We have provided the first functional characterization of this connection by combining optogenetic activation of BLA axons with whole-cell patch-clamp recordings of postsynaptic responses in CLA neurons. We found that light-evoked responses in CLA neurons were predominantly excitatory but were observed in only half of CLA cells. This suggests that BLA inputs selectively engage a subpopulation of CLA neurons. Moreover, the EPSCs produced in CLA neurons by BLA axon photostimulation were of relatively small amplitude, highly variable, and unreliable, even under conditions that were optimized for excitation (−70 mV holding potential in CLA neurons and wide-field photostimulation of many BLA axons). EPSPs evoked by photostimulating this pathway were similarly small in amplitude and well below the threshold for evoking APs.

EPSCs evoked in CLA neurons by BLA inputs were much smaller in amplitude (~5–55 pA) compared to those in neighboring AI L5b neurons (~15–100 pA). In contrast, photo- stimulation of ACC inputs can evoke EPSCs in the nA range in CLA projection neurons (and in interneurons) [29]. The coefficients of variation and failure probabilities of the EPSCs of CLA neurons were higher than those reported for many central synapses [55,56], implying that BLA input provides a noisy and inconsistent excitatory drive to individual CLA neurons and to the population as a whole.

To minimize confounds from short-term synaptic plasticity, we used a long inter-stimulus interval (5 s); only at much shorter inter-stimulus intervals (100 ms) is such plasticity evident (Figure 6). EPSC amplitudes appeared to vary randomly across trials, without systematic time-dependent trends, arguing against activity-dependent plasticity as the main source of variability. Because EPSC responses depend on effective opsin activation in presynaptic BLA axons, it remained formally possible that some failures reflected insufficient excitation of BLA axons. To exclude methodological explanations for these results, for example related to opsin expression or light delivery conditions, we performed parallel recordings from layer 5b neurons in the AI. This cortical structure receives dense BLA input [69]. In these cells, BLA-evoked EPSCs were larger, less variable, and far more reliable than in CLA neurons, confirming that our optogenetic stimulation robustly activated BLA axons. In addition, similar results were observed in the CLA when a second opsin, ChETA, was used to photostimulate BLA axons (Figure S5). Thus, the weak and noisy nature of BLA-to-CLA EPSCs appears to reflect a genuine property of synaptic transmission in this pathway.

Such properties imply that BLA inputs alone are insufficient to drive spiking in CLA neurons, a conclusion directly supported by our observations of small, subthreshold EPSPs produced in response to BLA axon photostimulation (Figure 6). Instead, we hypothesize that BLA input acts as a regulatory or “gating” signal that is excitatory yet must converge with other excitatory inputs to reach action potential firing threshold. In contrast, CLA inputs from some cortical regions appear to be much stronger and more reliable [29,40,70,71]. Taken together, our results suggest that BLA inputs are not a primary driver of CLA activity but instead must be integrated along with other inputs to drive AP firing in CLA neurons.

Such weak and variable excitatory inputs can still serve an important function. Even background synaptic “noise” can enhance sensitivity to other subthreshold signals, increase spike probability in a non-linear fashion, and support gain modulation [72,73,74,75,76,77]. Because the BLA is classically associated with valence and salience processing, we speculate that BLA input could, in principle, contribute subthreshold depolarizations that increase the likelihood that coincident cortical input drives CLA spiking. Our results therefore favor a model in which BLA projections provide a sparse, stochastic salience signal that influences—rather than determines—CLA activity. Further, the local inhibitory circuitry of the CLA is wired to ignore weak excitatory input—such as that from the BLA—until this input is paired with additional strong excitatory input [29]. In vivo recordings of CLA neuron activity will be needed to test such ideas.

### 4.3. BLA Functional Segregation Does Not Simply Follow Core–Shell Topology

Our results also revealed a functional heterogeneity of BLA input to CLA neurons, with only a subset of CLA neurons receiving BLA-driven EPSCs. We asked whether this functional heterogeneity could be explained by the striking anatomical enrichment of BLA axons in the CLA shell. Surprisingly, BLA functional connectivity was remarkably uniform across both the core and the shell: input probability, EPSC strength, variability, failure rate, and latency all were similar in both regions.

This dissociation between fiber density and synaptic drive indicates that macroscopic axon topology does not straightforwardly define which CLA projection neurons are functionally innervated by the BLA. Most strikingly, we observed clear BLA-evoked EPSCs in core neurons despite a low innervation of BLA fibers in the periphery of the core and its near absence in the center of the CLA core. This is complementary to the recent observation that input to the CLA from the anterior cingulate cortex, which is anatomically restricted to the CLA core [49,68], also excites both projection neurons and interneurons in the CLA shell [29]. Such structure–function mismatches suggest that the anatomical impression of sparse core innervation does not necessarily translate to synaptic function. Such observations also raise broader questions about other “shell-specific” inputs reported anatomically, such as from orbitofrontal or auditory cortex [49]. Systematic functional studies of other shell-biased inputs will be needed to determine whether BLA input to the CLA represents a special case or whether topology serves purposes beyond simple input segregation (e.g., controlling where on the dendritic tree inputs arrive, or which local circuits they preferentially recruit).

It is also possible that the dense BLA innervation of the CLA shell could preferentially target interneurons. Although interneurons represent only ~10% of neurons in the CLA [38], VIP and SST interneurons preferentially occupy the shell [38,66]. Thus, it is plausible that BLA axons in the shell target specific interneuron classes, such that the apparent core–shell difference in fiber density reflects differences in inhibitory microcircuit engagement rather than direct excitation of projection neurons. Future experiments will be needed to address this possibility.

### 4.4. Evidence for Monosynaptic BLA–CLA Connections

When examining circuit function, it is always valuable to distinguish monosynaptic and polysynaptic connections. For this purpose, we used TTX+4AP treatment [39,40,48]. The fact that EPSCs were detected in some (six out of 19) CLA neurons treated with TTX+4AP unequivocally demonstrated that some BLA–CLA connections are monosynaptic. This is consistent with previous transsynaptic tracing experiments demonstrating the existence of direct, monosynaptic BLA–CLA connections [31]. However, for technical reasons, it was difficult to conclude that polysynaptic BLA-to-CLA connections were responsible for the remaining 13 out of 19 experiments where EPSCs were not observed in TTX+4AP conditions. Poor signal-to-noise, particularly for the small, variable EPSCs we often observed when photostimulating BLA inputs, may make it difficult to detect the smaller EPSCs observed in TTX+4AP conditions (Figure 7 and Figure S3B). Another possibility is that some BLA–CLA synapses are “silent” at resting potentials—containing NMDA but few or no AMPA receptors—such that they would not be detected under our recording conditions but could be recruited during coincident depolarization [76]. Alternatively, it is possible that BLA input converges on a relatively small set of CLA projection neurons, which then make local, polysynaptic excitatory circuits [78]. Our results provide some indirect support for this possibility by revealing that the latency for EPSCs recorded in CLA neurons was more variable, and significantly longer on average, than for EPSCs in the AI (Figure 4J). These results would be expected if the CLA responses were a mix of monosynaptic and polysynaptic responses while the AI responses were monosynaptic.

### 4.5. Possible Mechanisms for Anatomy–Function Mismatches in BLA Innervation of CLA Core and Shell

One possible explanation for the striking mismatch between our histological and functional data is that the dendrites of CLA core neurons extend into the shell, potentially allowing shell-restricted BLA axons to synapse onto distal dendrites of core neurons. Filling CLA core neurons with neurobiotin revealed that they can extend dendrites radially into the CLA shell (Figure 2B). Given the shell-focused BLA fiber distribution (Figure 2D), this morphology suggests that BLA inputs innervate mainly distal dendrites of CLA core neurons. This could also explain the presence of monosynaptic BLA connections onto CLA core neurons despite the near absence of BLA fibers within the core. Future work that directly maps the distribution of BLA terminals on CLA neurons will be required to determine the precise location of synapses on CLA core neurons [39].

Several alternative mechanisms could also contribute. First, fiber fluorescence may not reflect synaptic terminal density: for example, regions with few labeled axons could still produce strong synaptic responses via high-probability synapses. Second, the location of the dendrites of CLA neurons and the plane of slicing may bias detection of BLA input, preventing our measurements from equally sampling synaptic connectivity across a neuron’s full dendritic arbor. Distinguishing these possibilities will likely require direct visualization of BLA synaptic terminals combined with more systematic dendritic sampling.

In contrast to projection neurons of the CLA core, the dendrites of CLA shell neurons were largely confined to the shell and avoided the core. Shell neurons also received less input from local PV interneurons, which were enriched in the core [29]. Such asymmetries suggest an information hierarchy, in which core neurons integrate inputs that terminate in both the core and the shell while shell neurons are restricted to shell-targeting inputs. Similar dendritic “cross-boundary” sampling has been proposed in other distributed cortical and subcortical systems [79,80]. In the superior colliculus (SC), superficial layer neurons only receive visual information, while deep layer neurons stretch their dendrites into the superficial layers to receive visual information for integration with other sensory modalities [79,81]. CLA core neurons may function in a similar manner, with such differences in input/dendritic organization potentially critical for integrating multiple input streams within the compact structure of the CLA.

### 4.6. BLA Inputs onto Topologically Distinct CLA Projection Neuron Populations

We also examined how BLA inputs are distributed across two topologically and functionally distinct output channels of the CLA: M1-projecting (*toM1*) and RSC-projecting (*toRSC*) neurons. These projection neuron populations occupy largely non-overlapping territories within the dCLA and CLA core, respectively, and participate in different large-scale networks [31,65].

Our anterograde tracing showed that BLA axons preferentially overlapped with neurons within the *toM1* zone in the dCLA, while BLA axon density was low for neurons within the *toRSC*-rich CLA core. This arrangement suggested that BLA inputs might influence the M1-related CLA network more strongly than the RSC-related network. This complements prior anatomical work on projection-specific CLA zones and suggests that different CLA output modules are differentially tuned to limbic drive. These anatomical observations also fit within a broader picture of CLA submodules associated with different cortical systems, including a dorsal module linked to motor networks (dCLA-to-M1), a CLA core linked to retrosplenial networks (CLA-to-RSC), and a dorsal endopiriform (DEn) region potentially linked to entorhinal–limbic circuits [65,68]. Our BLA tracing suggests that BLA input might be especially prominent in circuits associated with motor and limbic outputs, although we did not examine the function of inputs to the DEn.

Although our anatomical results suggested more BLA innervation of CLA neurons projecting to M1 than to those projecting to the RSC, in fact we observed very similar functional connectivity for both projection neuron types. This dissociation between axon density and functional connectivity again highlights the limitations of inferring synaptic influence from macroscopic anatomy alone. Labeling methods that do not directly label synapses are likely to show both synaptic terminations and passing fibers; thus, the dense BLA signal over *toM1* neurons may include axons that do not make synaptic contacts with those neurons or make only weak excitatory contacts. Similar to our observations with CLA core neurons, RSC-projecting neurons in the CLA core may extend dendrites into BLA-rich shell areas, whereas some M1-projecting neurons may reside in regions with many passing axons but few synapses. More generally, the complex dendritic and axonal topology of CLA neurons allows functional circuits to cut across gross anatomical boundaries.

## 5. Conclusions

Taken together, our results argue that the BLA-to-CLA pathway is anatomically selective but functionally weak and unreliable. BLA axons preferentially innervate the CLA shell, yet form comparable excitatory synapses with both core and shell neurons. Within projection-defined CLA neuron populations, while anatomical overlap suggests stronger BLA influence on M1-projecting neurons, functional connectivity with the BLA was similar for both projection neuron populations. These structure–function mismatches underscore that macroscopic anatomical maps—while essential—are not sufficient to predict how salience-related and valence-related information flows through CLA circuits.

Based on these findings, we propose that the BLA provides an excitatory input to the CLA that is subthreshold and noisy but capable of interacting with stronger cortical inputs. In this framework, the CLA receives BLA-encoded signals and integrates them with incoming signals to influence distributed cortical networks, including retrosplenial and motor systems. Future work should leverage these insights to determine how BLA–CLA circuitry works in vivo to shape behavior, particularly in contexts that require the integration of emotional salience with spatial, memory, or motor signals.

## Data Availability

The original contributions presented in this study are included in the article/Supplementary Materials. Further inquiries can be directed to the corresponding author.

## Supplementary Materials

The following supporting information can be downloaded at: https://www.mdpi.com/article/10.3390/cells15211937/s1, Figure S1: BLA fibers and cells projecting to BLA congregate in the CLA shell; Figure S2: Properties of light-evoked EPSCs did not differ between CLA projection neurons and interneurons; Figure S3. BLA-to-AI input is monosynapic; Figure S4. BLA fibers consistently overlap more with M1-projecting CLA neurons compared to RSC-projecting CLA neurons along the rostral-caudal axis; Figure S5. Light-evoked EPSC properties were similar when expressing ChrimsonR or ChETA in BLA axons.

## Abbreviations

The following abbreviations are used in this manuscript:

4AP	4-aminopyridine
AAV	Adeno-associated virus
ACSF	Artificial cerebrospinal fluid
AI	Agranular insula cortex
AMPA	α-amino-3-hydroxy-5-methyl-4-isoxazolepropionic acid
AP	Anterior–posterior
BLA	Basolateral amygdala
BLAFibers	BLA axons in the CLA with membrane-bound tdTomato
BLP	Basolateral amygdala—posterior
CLA	Claustrum
CV	Coefficient of variation
dCLA	Dorsal claustrum
DEn	Dorsal endopiriform nucleus
DI	Dysgranular insula
DV	Dorsal–ventral
EGFP	Enhanced green fluorescent protein
EPSC	Excitatory postsynaptic current
EPSP	Excitatory postsynaptic potential
IHC	Immunohistochemistry
IPSC	Inhibitory postsynaptic current
L5b	Layer 5b
M1	Primary motor cortex
mCh	mCherry
MD	Mediodorsal thalamus
ML	Medial–lateral
NMDA	N-methyl-D-aspartic acid
PBS	Phosphate-buffered saline
PFA	Paraformaldehyde
PFC	Prefrontal cortex
PV	Parvalbumin
ROI	Region of interest
RSC	Retrosplenial cortex
SD	Standard deviation
SST	Somatostatin
tDT	tdTomato
*toBLA*	CLA neurons projecting to the BLA
*toM1*	Neurons projecting to the M1
*toRSC*	Neurons projecting to the RSC
TTX	Tetrodotoxin
vCLA	Ventral claustrum
VIP	Vasoactive intestinal polypeptide
