# Supplementary material for: Basolateral Amygdala Input to the Claustrum: Topology, Excitatory Synapse Properties and Cellular Targets
Source: Cells. 2026 Oct 24;15(21):1937. doi: 10.3390/cells15211937 (PMC13762001; doi:10.3390/cells15211937)
Supplement: Supplementary file 1 [file cells-15-01937-s001.zip › cells-4580633-supplementary.pdf]

Supplementary Materials

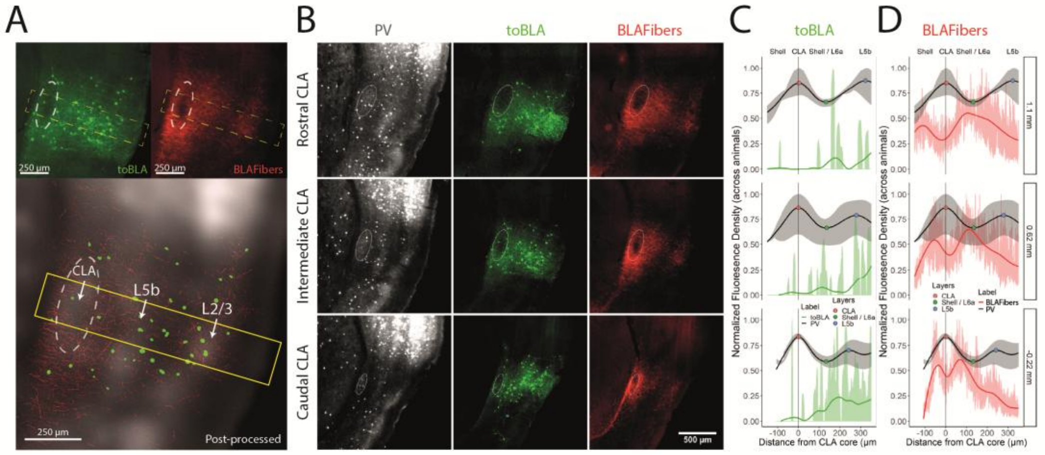

Figure S1. BLA fibers and cells projecting to BLA congregate in the CLA shell. (A-left) *toBLA* neurons are enriched in AI but sparse in the CLA core. (A-right) *BLAFibers* avoid the CLA core and densely innervate a surrounding shell and AI L2/3. (A-bottom) Composite post-processed image of (top panels & Fig 1A) used for unbiased quantification of layer enrichment. Filters were applied to segment somata, enhance fibers, and smooth PV signal (see Methods). Yellow box indicates the segment quantified in (Fig 1F). (B) Examples of BLA axons (*BLAFibers*, red), BLA projecting neurons (*toBLA*, green), and parvalbumin (PV, grey) immunohistochemistry along the rostral-caudal axis. White ellipse delineates CLA core. (C) Quantification of BLA projecting neurons (*toBLA*, green) and parvalbumin (PV, black) labelling across rostral (top), intermediate (middle), caudal (bottom) claustrum (n = 3). (D) Same as in (C), but with BLA axons (*BLAFibers*, red) (n = 3). (C&D) Bold foreground line represents average trace fit using local polynomial regression (LOESS). Raw values are displayed in background line. A lack of BLA-projecting soma (green) and reduced BLA fiber expression (red) in the CLA core is evident along the rostral-caudal axis. Enrichment of both viral tracers surrounding CLA core suggests the existence of a CLA shell.

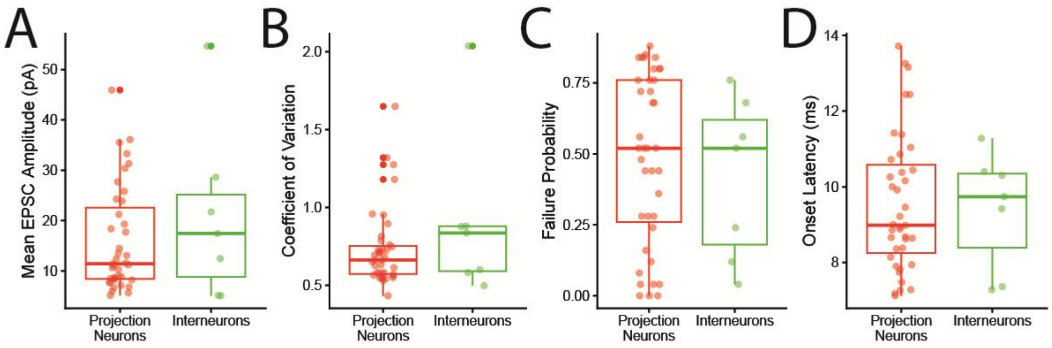

Figure S2. Properties of light-evoked EPSCs did not differ between CLA projection neurons and interneurons. (A-D) Comparison of EPSC responses properties: (A) Mean EPSC amplitude. (B) Coefficient of variation. (C) Failure Probability. (D) Onset Latency. Differences between projection neurons and interneurons were non-significant for all properties, based on Wilcoxon rank-sum test.

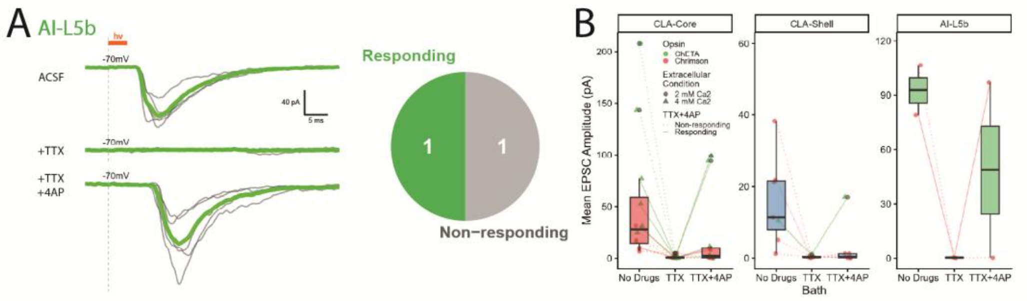

Figure S3. BLA-to-AI input is monosynapic. (A) Example EPSC recordings in a AI core neuron with a monosynaptic connection. Neuron was held at held at -70 mV holding potential (Vh). Traces (top to bottom): In ACSF, TTX or TTX+4AP. Grey lines represent raw traces and bold lines represent average EPSC. Orange bar represents timing of photostimulation (4 ms duration). (B) Mean light-induced EPSC response in different drug conditions, opsins and extracellular Ca^{2+}. Color represents different opsin conditions: ChETA (green) and ChrimsonR (red). Solid lines represent successful 4AP recovery and dotted lines represent unsuccessful recovery. Shapes represent extracellular Ca^{2+}: 2 mM (circle) and 4 mM (triangle). Extracellular Ca^{2+} was increased in a subset of TTX+4AP experiments to increase the likelihood of observing EPSCs in TTX+4AP conditions.

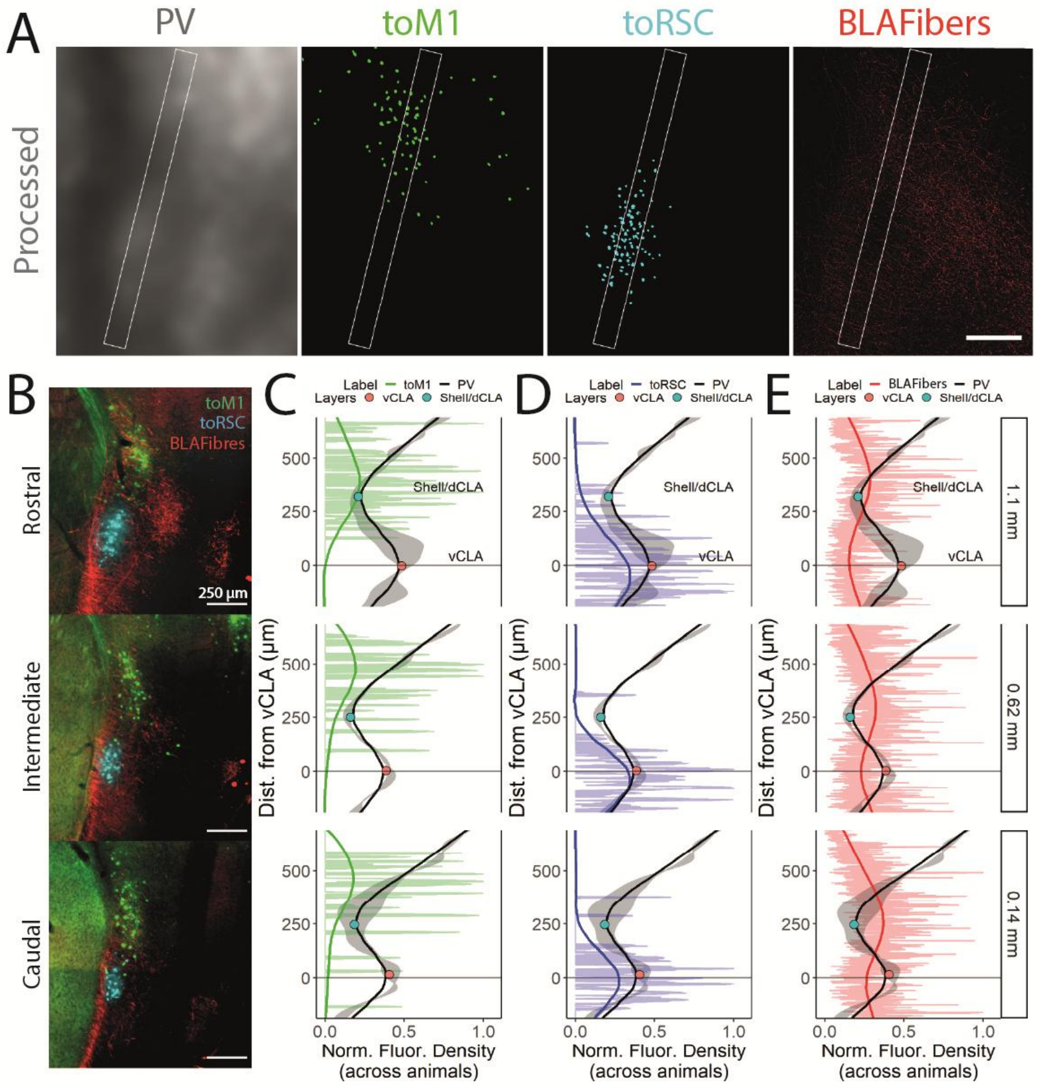

Figure S4. BLA fibers consistently overlap more with M1-projecting CLA neurons compared to RSC-projecting CLA neurons along the rostral-caudal axis. (A) Processed images from Fig 7C with profile plot region delineated in white. (B) Example composite images of M1 projecting neurons (*toM1*, green), RSC projecting neurons (*toRSC*, cyan), and BLA axons (*BLAFibers*, red) along the rostral-caudal axis. (C-E) Profile quantification of fluorescent channels in (B) coupled with parvalbumin (PV, black) labelling across rostral (top), intermediate (middle), caudal (bottom) claustrum (n = 2). Profile was taken in accordance to (Figure 8C) (C-E) Bold foreground line represents average trace fit using local polynomial regression (LOESS). Raw values are displayed in background line. (C) For M1 projecting neurons (*toM1*, green). (D) For RSC projecting neurons (*toRSC*, cyan). (E) For BLA axons (*BLAFibers*, red).

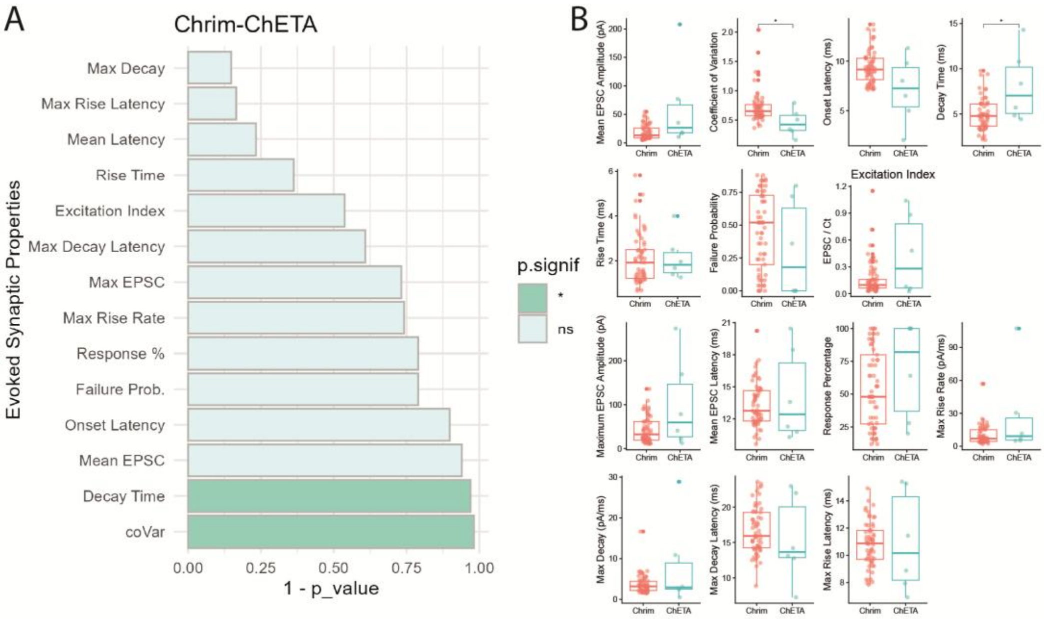

Figure S5. Light-evoked EPSC properties were similar when expressing ChrimsonR or ChETA in BLA axons. (A) p-value comparison of properties relating to EPSC dynamics between ChrimsonR and ChETA: Significance testing was based on Wilcoxon rank-sum test. (B) Plots for each of the properties tested in (A). Differences were non-significant in all properties related to EPSC dynamics, except Decay time and coVar.
